# Complexoform-restricted covalent TRMT112 ligands that allosterically agonize METTL5

**DOI:** 10.1038/s41589-025-02099-5

**Published:** 2026-01-08

**Authors:** F. Wieland Goetzke, Steffen M. Bernard, Cheng-Wei Ju, Jonathan Pollock, Kristen E. DeMeester, Jacob Gross, Gabriel M. Simon, Chuan He, Bruno Melillo, Benjamin F. Cravatt

**Affiliations:** 1 Department of Chemistry, Scripps Research, La Jolla, CA USA; 2 Vividion Therapeutics, San Diego, CA USA; 3 Department of Chemistry, The University of Chicago, Chicago, IL USA; 4 Department of Biochemistry and Molecular Biology, The University of Chicago, Chicago, IL USA; 5 Pritzker School of Molecular Engineering, The University of Chicago, Chicago, IL USA; 6 Howard Hughes Medical Institute, The University of Chicago, Chicago, IL USA

**Keywords:** Chemical modification, Proteomics, X-ray crystallography, Small molecules, Post-translational modifications

## Abstract

Adaptors serve as hubs to regulate diverse protein complexes in cells. This multitude of functions can complicate the study of adaptors, as their genetic disruption may simultaneously impair the activities of several compositionally distinct complexes (or adaptor ‘complexoforms’). Here we describe the chemical proteomic discovery of bicyclopyrrolidine acrylamide stereoprobes that react with C100 of the methyltransferase (MT) adaptor TRMT112 in human cells. Curiously, the stereoprobes showed negligible reactivity with uncomplexed recombinant TRMT112 and we found that this interaction was restored exclusively in the presence of METTL5 but not other MTs. A cocrystal structure revealed stereoprobe binding to a composite pocket proximal to C100 of TRMT112 that is templated by METTL5 and absent in other TRMT112:MT complexes. Structural rearrangements promoted by stereoprobe binding in turn lead to allosteric agonism of METTL5, thus revealing how covalent ligands targeting a pleiotropic adaptor can confer partner-specific functional effects through reactivity with a single complexoform.

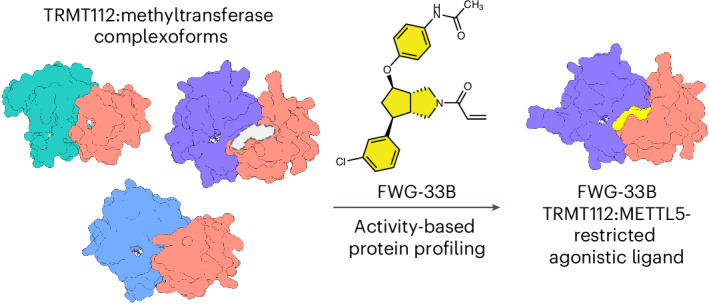

## Abstract (web summary)

Activity-based protein profiling identifies covalent small molecules that potentiate the activity of the METTL5:TRMT112 complex through binding to a complexoform-restricted allosteric pocket absent in other TRMT112:methyltransferase complexes

## Main

The ~20,000 protein-encoding genes in the human genome yield a much larger number of proteoforms generated by myriad post-transcriptional and post-translational mechanisms^1–4^. Major categories of proteoforms include splice variants (spliceoforms), covalently modified proteins (for example, phosphorylated or glycosylated proteins) and protein complexes of differing compositions (complexoforms^5,6^). Complexoforms, in particular, have been defined as ‘protein complexes formed by monomeric proteoform arrangements of one or more gene products^5^. Such diversification of the proteome provides important layers of control over dynamic metabolic and signaling pathways in the cell^1–4^.

Small molecules serve as central tools for perturbing the functions of proteins in cells and there is growing interest in the discovery of chemical probes that target specific proteoforms of proteins^7–12^. Such proteoform-restricted probes have the potential to modulate biology with more precision than that achieved by the complete pharmacological or genetic disruption of proteins. Experimental approaches for identifying ligands that bind specific proteoforms, however, remain poorly defined. These efforts are challenged by the diverse and dynamic states of proteins in cells, only a small subset of which are typically accounted for in conventional screening assays performed with recombinant proteins.

We and others have shown that activity-based protein profiling (ABPP) offers a versatile chemical proteomic approach for globally mapping small molecule–protein interactions in human cells^13–16^. ABPP of focused libraries of stereochemically defined covalent (electrophilic or photoreactive) small molecules (or ‘stereoprobes’) has been particularly useful for identifying cryptic sites of ligandability on diverse classes of proteins, including adaptors, RNA-binding proteins and transcriptional regulators^17–22^. Some of these small molecule–protein interactions require additional factors for recapitulation in recombinant systems (for example, the presence of DNA for stereoprobe binding to a transcription factor^19^), thus underscoring the potential for ABPP to illuminate state-dependent protein liganding events in cells.

The stereoprobes studied to date include both fragments^17,22^ and more elaborated compounds bearing sp^3^-rich, entropically constrained cores^18,20,21,23,24^. This work has underscored the importance of scaffold diversity for expanding the ligandability of the human proteome, as different classes of acrylamide stereoprobes (azetidines^20,24^, tryptolines^18,20,23^ and spirocycles^21^) show only limited overlap in their protein interactions in cells. Here, we describe the synthesis of bicyclopyrrolidine acrylamide (BcpAc) stereoprobes and their analysis by cysteine-directed and protein-directed ABPP^23^. We show that these compounds, despite having substantially attenuated intrinsic and proteomic reactivity compared with other classes of acrylamide stereoprobes, stereoselectively engage a unique set of proteins in human cancer cells. Integration of cysteine-directed and protein-directed ABPP data revealed a surprising inconsistency in the apparent reactivity of BcpAcs with the methyltransferase (MT) adaptor protein TRMT112 that we determined to reflect the exclusive engagement of C100 when this adaptor is bound to METTL5 but not other MT partners. Structural studies reveal that the BcpAcs bind to a composite pocket at the TRMT112:METTL5 interface in close proximity to C100 that is not found in other TRMT112:MT complexes. We finally show that BcpAcs promote allosteric changes in the TRMT112:METTL5 complex that correlate with enhanced RNA MT activity. These findings provide a chemical proteomic roadmap for the discovery of complexoform-restricted covalent liganding events and reveal how such interactions can yield selective agonists of one partner (METTL5) of a pleiotropic adaptor protein (TRMT112).

## Results

### Design and synthesis of BcpAc stereoprobes

**Fig. 1:**
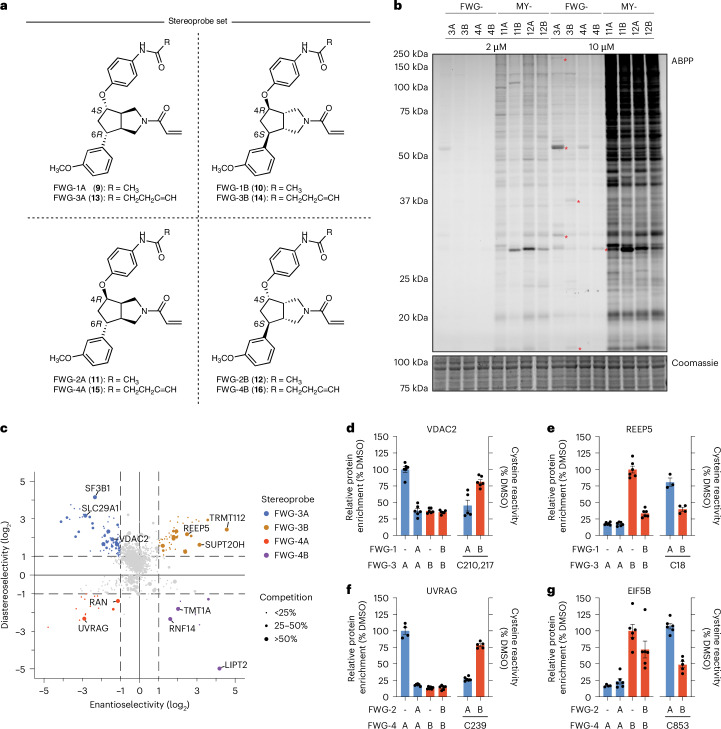
Structures and chemical proteomic analysis of BcpAc stereoprobes. **a**, Structures of non-alkyne (FWG-1A (**9**), FWG-1B (**10**), FWG-2A (**11**) and FWG-2B (**12**)) and alkyne (FWG-3A (**13**), FWG-3B (**14**), FWG-4A (**15**) and FWG-4B (**16**)) BcpAc stereoprobes. **b**, Gel-ABPP data for Ramos cells treated with the indicated concentrations of alkyne BcpAc or azetidine acrylamide^24,30^ (MY-11A, MY-11B, MY-12A and MY-12B; Supplementary Fig. 1) stereoprobes for 1 h. Stereoprobe-reactive proteins were visualized by CuAAC conjugation to an azide-rhodamine reporter group, SDS–PAGE and in-gel fluorescence scanning. The gels show ABPP data (top) and Coomassie blue staining (bottom). Red asterisks mark representative proteins that were stereoselectively engaged by BcpAcs (shown for 10 µM condition). Data are from a single experiment representative of two independent experiments. **c**, Quadrant plot showing stereoselectively liganded proteins for each stereoconfiguration of the BcpAcs in protein-directed ABPP experiments. Ramos cells were treated with non-alkyne competitors FWG-1A, FWG-1B, FWG-2A and FWG-2B (50 μM, 3 h), followed by stereomatched alkynes FWG-3A, FWG-3B, FWG-4A and FWG-4B (10 μM, 1 h) and protein-directed ABPP analysis. Enantioselectivity (*x* axis) is the ratio of enrichment for one stereoisomer versus its enantiomer and diastereoselectivity (*y* axis) is the ratio of enrichment of one stereoisomer versus the average of its two diastereomers. Data are average values for 4–6 independent biological experiments. **d**–**g**, Bar graphs comparing cysteine-directed and protein-directed ABPP for representative proteins stereoselectively liganded by BcpAcs (VDAC2 (**d**), REEP5 (**e**), UVRAG (**f**) and EIF5B (**g**); complete list of liganded proteins in Table 1). Data are average values ± s.e.m. for 3–6 independent biological experiments. Source data

**Extended Data Fig. 1:**
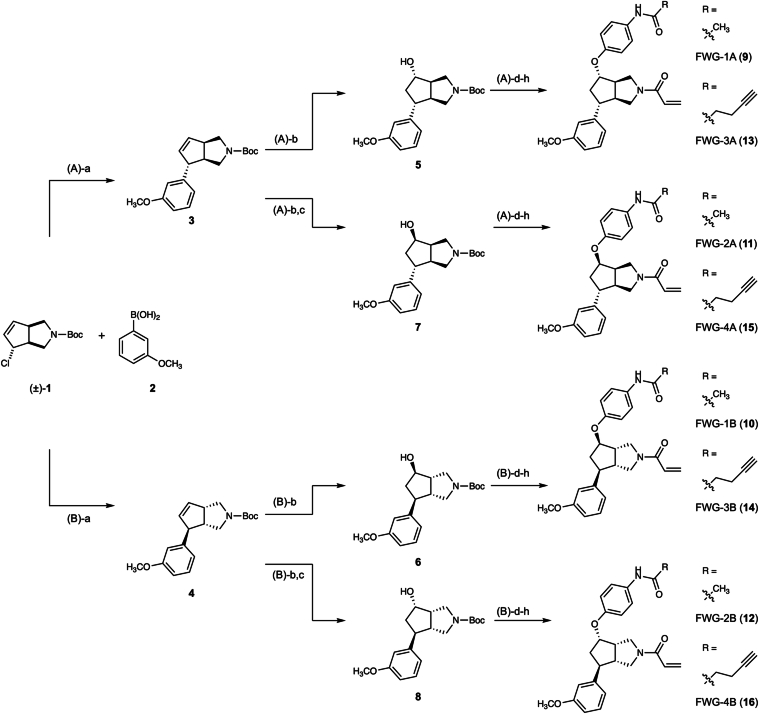
Scheme for the catalytic stereodivergent synthesis of bicyclopyrrolidine acrylamide (BcpAc) stereoprobes. *General conditions* (representative for **FWG-3A** and **FWG-4A**; also see Supplementary Information): **a**. 3-H_3_CO-Ph-B(OH)_2_, aq. CsOH, (*S*)-SEGPHOS (6 mol%), [Rh(cod)OH]_2_ (2.5 mol%), THF/H_2_O, 65 °C, overnight, 75%, 99% ee; **b**. HBpin, Cs_2_CO_3_, [Rh(PPh_3_)_3_Cl] (10 mol%), THF, r.t., overnight, then H_2_O_2_, aq. NaOH, 0 °C, 1 h, 51%; **c**. (1) 4-NO_2_-Ph-CO_2_H, PPh_3_, DIAD, THF, 0 °C to r.t., overnight, (2) K_2_CO_3_, MeOH/H_2_O, r.t., overnight, 68%; for FWG-3A: **d**. 4-NO_2_-Ph-F, KHMDS, PhCH_3_/DMF, 0 °C to r.t., overnight, 80%; **e**. H_2_, Pd/C, MeOH, 3 h, r.t., 88%; **f**. HC≡C(CH)_2_CO_2_H, HATU, DIPEA, CH_2_Cl_2_, r.t., overnight (no purification); **g**. TFA, CH_2_Cl_2_, r.t., 1 h (no purification); **h**. acryloyl chloride, DIPEA, CH_2_Cl_2_, r.t., 2 **h**, 39% (over 3 steps); for FWG-4A: **d**. 4-NO_2_-Ph-F, KHMDS, PhCH_3_/DMF, 0 °C to r.t., overnight, 33%; **e**. H_2_, Pd/C, MeOH, 3 h, r.t., 88%; **f**. HC≡C(CH)_2_CO_2_H, HATU, DIPEA, CH_2_Cl_2_, r.t., overnight (no purification); **g**. TFA, CH_2_Cl_2_, r.t., 1 h (no purification); **h**. acryloyl chloride, DIPEA, EtOAc, r.t., 2 h, 64% (over 3 steps). *Abbreviations:* cod = 1,5-cyclooctadiene; HATU = hexafluorophosphate azabenzotriazole tetramethyl uranium; DIAD = diisopropyl azodicarboxylate; DIPEA = *N,N*-diisopropylethylamine; DMF = *N,N*-dimethylformamide; KHMDS = potassium bis(trimethylsilyl)amide; pin = pinacolato; r.t. = room temperature; TFA = trifluoroacetic acid; THF = tetrahydrofuran.

Bicyclic and spirocyclic saturated heterocycles have emerged as building blocks of interest for medicinal chemistry because their high sp^3^ content and conformational restriction can offer beneficial (physio)chemical properties for binding to challenging (for example, shallow and cryptic) protein pockets^25,26^. Here, we designed and synthesized two diastereoisomeric pairs of enantiomers of acrylamide stereoprobes based on an octahydrocyclopenta[c]pyrrole core scaffold (henceforth referred to as BcpAcs) (Fig. 1a). The structures feature two diversifiable appendages and an endocyclic nitrogen as an attachment point for a cysteine-reactive acrylamide. The synthesis initiates with an enantioselective (>95% e.e.) rhodium-catalyzed cross-coupling between a racemic allylic chloride ((±)-**1**)^27^ and an arylboronic acid (**2**). The resulting products (**3**, **4**) are then subjected to a diastereoselective and regioselective rhodium-catalyzed hydroboration–oxidation sequence (**5**, **6**)^28^ with an optional Mitsunobu stereoinversion of the secondary alcohol to access the alternative diastereomers (**7**, **8**) (Extended Data Fig. 1). Each stereoisomer can then be elaborated into an acrylamide in a synthetic sequence including S_N_Ar reactions with electron-deficient aryl halides^29^, additional functionalization reactions and a final deprotection–acryloylation sequence.

### ABPP of BcpAc stereoprobes in human cancer cells

**Extended Data Fig. 2:**
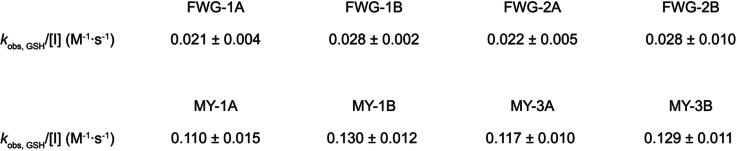
Glutathione (GSH) reactivity of stereoprobes. Glutathione (GSH) reactivity measurements for FWG-1A/B and FWG-2A/B compared to previously described azetidine acrylamides MY-1A/B and MY-3A/B (see Supplementary Fig. 1). Data are average values ± s.e.m. for three independent experiments. [I] = inhibitor concentration. Source data

Each pair of enantiomeric BcpAc stereoprobes was synthesized in non-alkyne (FWG-1A (**9**), FWG-1B (**10**), FWG-2A (**11**) and FWG-2B (**12**)) and alkyne (FWG-3A (**13**), FWG-3B (**14**), FWG-4A (**15**) and FWG-4B (**16**)) form (Fig. 1a) for use in gel and mass spectrometry (MS) ABPP experiments in the Ramos B-lymphocyte human cancer cell line. Gel-ABPP experiments revealed that the four alkynylated BcpAcs produced several stereoselective protein engagement events in Ramos cells (Fig. 1b, red asterisks) against a background of much lower overall proteomic reactivity compared with previously reported azetidine acrylamides (MY-1A/B and MY-3A/B)^24,30^ (Fig. 1b). The observed proteomic reactivity profiles matched the respective glutathione (GSH) reactivities of each stereoprobe class (Extended Data Fig. 2).

**Extended Data Fig. 3:**
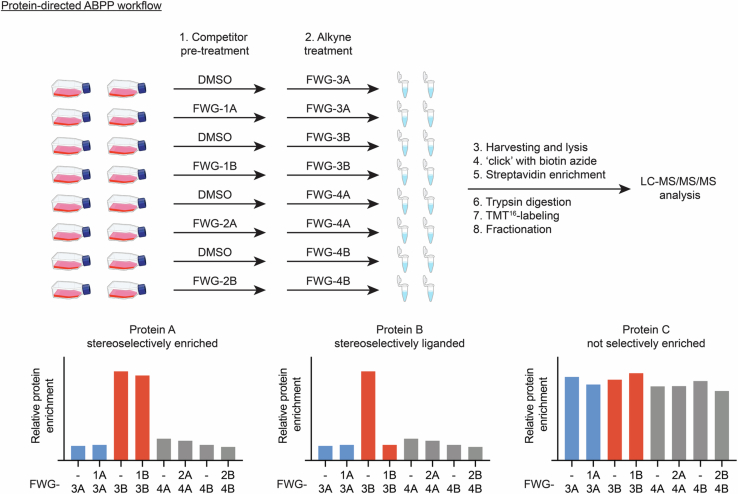
General workflow for quantifying stereoselectively liganded proteins by protein-directed ABPP performed with BcpAc stereoprobes. Multiplexed format (TMT 16plex) for protein-directed ABPP experiments (performed as described previously^23^) for quantifying stereoselective enrichment of proteins by alkyne stereoprobes FWG-3A, 3B, 4A, and 4B, and the competitive blockade of this enrichment by non-alkynes FWG-1A, 1B, 2A and 2B. Two replicates of each group are generated per TMT 16plex experiment. Mock profiles of proteins showing stereoselectively enrichment, but not competition (protein A), stereoselective enrichment and competition (protein B), and neither stereoselective enrichment or competition (protein C).

We next analyzed the BcpAcs by protein-directed ABPP^23^ (Extended Data Fig. 3), where Ramos cells were first treated with non-alkyne ‘competitors’ (FWG-1A, FWG-1B, FWG-2A and FWG-2B, 50 µM, 3 h or DMSO control), followed by stereochemically matched alkynes (FWG-3A, FWG-3B, FWG-4A and FWG-4B, 10 µM, 1 h). Cells were then lysed and alkyne-modified proteins were conjugated to biotin-azide by copper-catalyzed azide–alkyne cycloaddition (CuAAC or click) chemistry^31,32^, enriched using streptavidin beads, trypsinized and analyzed by multiplexed (tandem mass tag (TMT)) MS-based proteomics. A total of 20 proteins were stereoselectively liganded by the BcpAcs according to the following criteria: more than twofold enantioselective enrichment by an alkyne BcpAc and >50% competitive blockade of this enrichment by the corresponding non-alkyne competitor (Table 1 and Supplementary Data 1). A threshold of >50% competition for assignment of liganded proteins was chosen because (1) smaller decreases (<50%) were more challenging to interpret with confidence by TMT quantification in our MS-based proteomic experiments and (2) we aimed to prioritize robust stereoprobe–protein interactions for downstream functional studies. As visualized in a quadrant plot display (Fig. 1c), the stereoselectively liganded proteins were distributed across the four BcpAcs with a slight bias toward the stereoprobes with a *cis* display of appendages (FWG-1A/3A (4*S*,6*R*) and FWG-1B/3B (4*R*,6*S*)).

**Table 1:** List of proteins that were stereoselectively liganded by BcpAc stereoprobes as determined by cysteine- and protein-directed ABPP of Ramos cells (a protein was considered liganded if it displayed >2-fold enantioselective enrichment by 1 or more stereoprobe in protein-directed ABPP experiments, and >50% competition of this enrichment by non-alkyne stereoprobes in cysteine and/or protein-directed ABPP experiments)

Protein name	Accession code (UniProt)	Protein class	Active stereoprobes	Competition (%)	Cysteine-directed ABPP	Targets of other electrophilic stereoprobes^20,21,23,24^
TBC1D31	Q96DN5	Adaptor	FWG-1B/3B	>90		
TRMT112	Q9UI30	Adaptor	FWG-1B/3B	>90		
RNF14	Q9UBS8	E3 ligase	FWG-2B/4B	>80	C212^a^	
STK39	Q9UEW8	Kinase	FWG-1A/3A	>80		Y (C334)
TARBP1	Q13395	MT	FWG-1B/3B	>80		
TMT1A	Q9H8H3	MT	FWG-2B/4B	>80		
UVRAG	Q9P2Y5	Uncategorized	FWG-2A/4A	>80	C239	Y (C239)
SF3B1	O75533	Splicing factor	FWG-1A/3A	>70		Y (C1111)
SLC29A1	Q99808	Transporter	FWG-1A/3A	>70		Y
ALDH2	P05091	Dehydrogenase	FWG-1A/3A	>60		
LIPT2	A6NK58	Lipoyltransferase	FWG-2B/4B	>60		
REEP5	Q00765	Uncategorized	FWG-1B/3B	>60	C18	Y (C18)
SLCO4A1	Q96BD0	Transporter	FWG-2B/4B	>60		
SUPT20H	Q8NEM7	Transcription factor	FWG-1B/3B	>60		
VDAC2	P45880	Ion channel	FWG-1A/3A	>60	C210,227	
ATF7IP	Q6VMQ6	Transcription factor	FWG-1B/3B	>50		
DNAJA1	P31689	Chaperone	FWG-1B	>50	C394	
EIF5B	O60841	GTPase	FWG-2B	>50	C853	
HERC2	O95714	E3 ligase	FWG-1B	>50	C1005	
RAN	P62826	GTPase	FWG-2A/4A	>50		
TXNDC15	Q96J42	Uncategorized	FWG-1B/3B	>50		Y
TMX1	Q9H3N1	Redox enzyme	FWG-1A/3A	>50		Y (C56)
TM9SF2	Q99805	Transporter	FWG-1B/3B	>50		

Protein class assignments are from Gene Ontology (GO; Panther), KEGG BRITE and UniProt. Y, yes. ^a^With Glu-C digestion.

We next performed cysteine-directed ABPP experiments^9,18,33,34^ that quantified, in total, >18,000 cysteines in Ramos cells. Six of these cysteines showed stereoselective liganding by the BcpAcs, defined as >50% enantioselective decreases in iodoacetamide-desthiobiotin (IA-DTB) reactivity (Table 1 and Supplementary Data 1). In general, cysteine-directed and protein-directed ABPP measured similar extents of BcpAc engagement for the protein targets quantified by both methods (Fig. 1d–g).

The 23 total protein targets of the BcpAcs originated from diverse structural and functional classes and included several proteins that have not yet been liganded by other classes of acrylamide stereoprobes^20,21,23,24^ and for which chemical tools are more generally lacking (Table 1). Our ABPP data, thus, indicated that BcpAcs engage a unique set of proteins in human cells and do so against a backdrop of reduced overall proteomic reactivity compared with other classes of electrophilic stereoprobes.

### Initial characterization of BcpAc-liganded proteins

**Extended Data Fig. 4:**
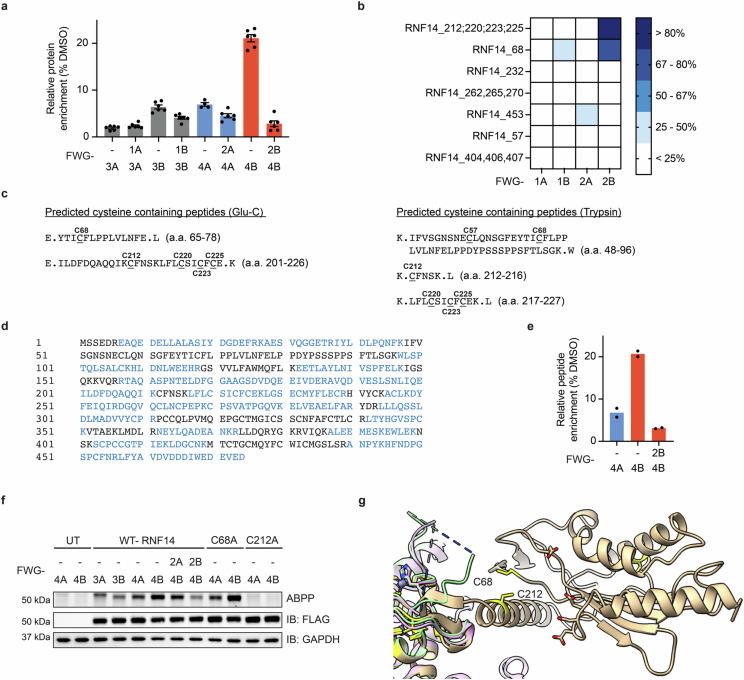
Mapping cysteine-212 (C212) as the site of BcpAc engagement in RNF14. **a**, Bar graph showing protein-directed ABPP data for RNF14 (from experiments summarized in Fig. 1c). Data are average values ± s.e.m. for 4–6 independent biological experiments. **b**, Heatmap showing RNF14 peptides from cysteine-directed ABPP experiments performed on HEK293T cells recombinantly expressing RNF14 and using a Glu-C protease digestion protocol. The RNF14_C212,220,223,225 and RNF14_C68 peptides showed stereoselective blockade of IA-DTB enrichment by FWG-2B. HEK293T cells were transfected with wild-type (WT)-RNF14, treated with FWG-1A, 1B, 2A, and 2B (50 μM, 3 h), followed by lysis and treatment with IA-DTB (100 μM, 1 h) and cysteine-directed ABPP with Glu-C digestion. Data are average values ± s.e.m. for two independent biological experiments. **c**, Predicted RNF14 peptides containing C212,220,223,225 and C68 from Glu-C and trypsin digestion. a.a. = amino acids. **d**. Amino acid sequence of RNF14 highlighting in blue the tryptic peptide sequences that were quantified by protein-directed ABPP experiments performed on HEK293T cells recombinantly expressing WT-RNF14 and treated with FWG-1A, 1B, 2A, and 2B (50 μM, 3 h), followed by stereomatched alkynes FWG-3A, 3B, 4A, and 4B (10 μM,1 h). Data are from two independent biological experiments. **e**, Stereoselective enrichment of the RNF14_220,223,225 tryptic peptide in FWG-4B-treated cells from protein-directed ABPP experiments performed on HEK293T cells recombinantly expressing WT-RNF14 (for treatment conditions see Extended Data Fig. 4d). Data are average values for two independent biological experiments. **f**, Gel-ABPP data demonstrating stereoselective and site-specific engagement of recombinant WT-RNF14-FLAG and C68A-RNF14-FLAG, but not C212A-RNF14-FLAG by FWG-4B. HEK293T cells expressing the indicated recombinant RNF14 proteins were pre-treated with non-alkyne competitors FWG-2A and 2B (50 μM, 3 h), followed by FWG-3A, 3B, 4A, and 4B (10 μM, 1 h), and analyzed by gel-ABPP (as described in Fig. 1b). Data are from a single experiment representative of two independent experiments. IB = immunoblot; UT = untransfected. **g**, AlphaFold model of RNF14 predicting proximity between C68 and C212 (8 Å). RNF14_C212 is on the N-terminal side of the RNF14 RBR/TRIAD supradomain. The alignment of the TRIAD supradomain of RNF14 with determined TRIAD1 structures in green and pink (PDB: 7ONI and PDB: 7OD1). Source data

A substantial number of BcpAc-liganded proteins were identified by protein-directed but not cysteine-directed ABPP. As described previously^23^, such liganding events often occur at cysteines residing on nonproteotypic peptides that are difficult to detect in cysteine-directed ABPP experiments. For the E3 ubiquitin ligase RNF14—a target of the FWG-2B/4B stereoprobes (Extended Data Fig. 4a)—we succeeded in mapping the site of liganding as C212 by a combination of cysteine-directed and protein-directed ABPP experiments performed on cells recombinantly expressing this protein. For the cysteine-directed ABPP experiments, we used an alternative Glu-C protease digest, which quantified seven cysteine-containing peptides in RNF14, two of which showed stereoselective decreases in IA-DTB reactivity in FWG-2B-treated cells: amino acids 65–78 (containing C68) and 201–226 (containing C212, C220, C223 and C225) (Extended Data Fig. 4b,c and Supplementary Data 1). Complementary protein-directed ABPP experiments performed with trypsin digests identified a stereoselectively enriched and unmodified peptide containing C220, C223 and C225 in FWG-4B-treated cells (Extended Data Fig. 4d,e and Supplementary Data 1), indicating that these cysteines are not the site of BcpAc reactivity. Subsequent gel-ABPP experiments revealed that the RNF14-C68A mutant maintained stereoselective reactivity with FWG-4B, whereas the RNF14-C212A mutant lost reactivity (Extended Data Fig. 4f), supporting that C212 is the site of engagement by BcpAcs. Interestingly, the AlphaFold^35,36^-predicted structure of RNF14 places C68 and C212 in close proximity (Extended Data Fig. 4g), suggesting that FWG-2B reactivity with C212 may sterically impair IA-DTB reactivity with C68.

**Fig. 2:**
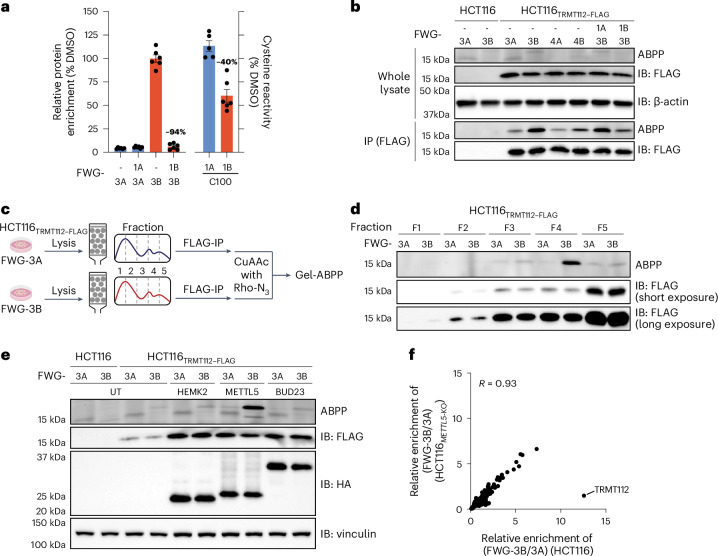
BcpAcs engage a TRMT112:METTL5 complexoform in cells. **a**, Bar graphs comparing cysteine-directed and protein-directed ABPP for TRMT112 (bar graphs including all stereoisomers in Extended Data Fig. 4a,b). Left *y* axis: protein-directed ABPP data for Ramos cells treated with DMSO and non-alkyne competitors FWG-1A and FWG-1B (50 μM, 3 h), followed by stereomatched alkynes FWG-3A and FWG-3B (10 μM, 1 h). Right *y* axis: cysteine-directed ABPP data for Ramos cells treated with FWG-1A and FWG-1B (50 μM, 3 h), followed by lysis and treatment with IA-DTB (100 μM, 1 h). Data are average values ± s.e.m. for 5–6 independent biological experiments. **b**, Gel-ABPP data demonstrating stereoselective engagement of recombinant TRMT112–FLAG by FWG-3B visible after anti-FLAG IP. HCT116 (parental) and HCT116_TRMT112__–FLAG_ cells were treated with FWG-1A and FWG-1B (50 μM, 3 h), followed by treatment with FWG-3A, FWG-3B, FWG-4A and FWG-4B (10 μM, 1 h). TRMT112–FLAG was enriched by IP with anti-FLAG magnetic beads and analyzed by gel-ABPP (as described in Fig. 1b). Data are from a single experiment representative of two independent experiments. IB, immunoblot. **c**, Workflow of the SEC–ABPP experiments (Methods). **d**, Gel-ABPP (top) and western blotting (middle and bottom) data of SEC fractions from HCT116_TRMT112__–FLAG_ cells treated with FWG-3A or FWG-3B (10 µM, 1 h) showing that the FWG-3B-reactive proteoform of TRMT112 elutes in fraction 4 while the majority of the TRMT112 protein is found in fraction 5. Data are from a single experiment representative of two independent experiments. **e**, Gel-ABPP data demonstrating stereoselective engagement of TRMT112 by FWG-3B in HCT116_TRMT112__–FLAG_ cells coexpressing METTL5 but not HEMK2 or BUD23. HCT116_TRMT112__–FLAG_ cells were transiently transfected with complementary DNA for HA-tagged HEMK2, METTL5 and BUD23, followed by treatment with FWG-3A and FWG-3B (10 μM, 1 h) and gel-ABPP analysis. Data are from a single experiment representative of two independent experiments. **f**, Protein-directed ABPP experiments demonstrating loss of stereoselective enrichment of TRMT112 by FWG-3B in *METTL5*-KO cells. Scatter plot comparing the stereoselective enrichment profiles (FWG-3B/FWG-3A; 10 µM, 1 h) of proteins in parental (*x* axis) versus *METTL5*-KO (*y* axis) HCT116 cells. Data are average values for four independent biological experiments. The linear regression analysis was performed excluding the TRMT112 data. Source data

**Extended Data Fig. 5:**
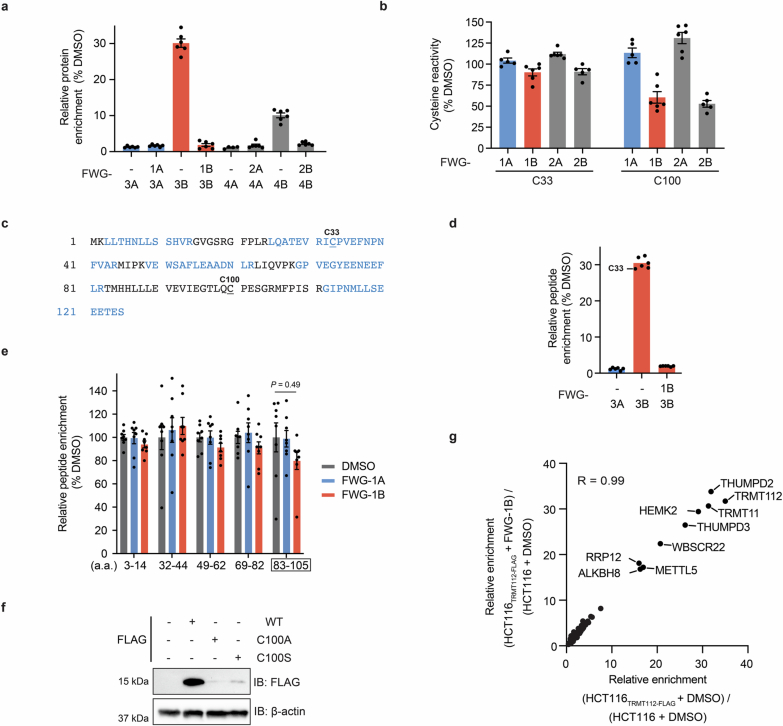
BcpAc stereoprobes engage a minor TRMT112 proteoform in cells. **a**, Protein-directed ABPP data for TRMT112 from Ramos cells treated with FWG-1A, 1B, 2A, and 2B (50 μM, 3 h), followed by stereomatched alkynes FWG-3A, 3B, 4A, and 4B (10 μM,1 h). **b**, Cysteine-directed ABPP data for TRMT112_C33 and C100 from Ramos cells treated with FWG-1A, 1B, 2A, and FWG-2B (50 μM, 3 h). For **a**, **b**, data are average values ± s.e.m. for 4–6 independent biological experiments. **c**, Amino acid sequence of TRMT112. Amino acids in blue were quantified by protein-directed ABPP. **d**, Quantification of TRMT112 tryptic peptides, including the C33-containing peptide by protein-directed ABPP. **e**, Quantification of the indicated tryptic peptides for TRMT112 from anti-FLAG IP-MS experiments performed with HCT116_TRMT112-FLAG_ cells treated with DMSO or FWG-1A or FWG-1B (50 µM, 4 h). Data are average values ± s.e.m. normalized to DMSO for eight independent biological experiments. a.a. = amino acids. Statistical significance between the DMSO and the FWG-1B treated peptides was assessed using two-sided, unpaired-multiple *t*-tests (Holm-Šídák approach to multiple comparisons; reported *P* values are adjusted *P* values). **f**, Western blot showing negligible expression of C100A- and C100S-TRMT112 mutants relative to wild-type (WT)-TRMT112 in HCT116 cells. Data are from a single experiment representative of two independent experiments; IB = immunoblot. **g**, Proteins co-enriched with TRMT112 in anti-FLAG IP-MS experiments performed with parental HCT116 cells (control) or HCT116_TRMT112-FLAG_ cells treated with DMSO of FWG-1B (50 µM, 4 h). Scatter-plot showing strong correlation (R = 0.99) for co-enriched proteins in DMSO- (*x*-axis) vs FWG-1B- (*y*-axis) treated HCT116_TRMT112-FLAG_ cells. Data are average values for eight independent biological experiments. Source data

A deeper analysis of our chemical proteomic data suggested that a different mechanism may underlie the exclusive mapping of TRMT112 as a target of BcpAcs by protein-directed but not cysteine-directed ABPP. TRMT112 is an essential 15-kDa adaptor protein that binds to and regulates the function of a diverse set of *S*-adenosylmethionine (SAM)-dependent MTs involved in RNA, DNA and protein methylation^37–44^. Protein-directed ABPP revealed that each pair of BcpAcs showed enantioselective reactivity with TRMT112, with FWG-1B/3B showing the strongest engagement profile followed by FWG-2B/4B (Fig. 2a and Extended Data Fig. 5a). Both cysteines in TRMT112 (C33 and C100) were quantified by cysteine-directed ABPP, with C100 showing a modest (~40%) stereoselective decrease in IA-DTB reactivity in cells treated with FWG-1B and FWG-2B (Fig. 2a and Extended Data Fig. 5b). This change, however, was much smaller than the >90% blockade of TRMT112 enrichment caused by FWG-1B in protein-directed ABPP experiments (Fig. 2a). As additional evidence that C100 was the likely site of BcpAc engagement in TRMT112, the tryptic peptide containing this cysteine was not observed in our protein-directed ABPP experiments (Extended Data Fig. 5c,d).

Curious about the quantitative discrepancy in liganding of TRMT112 as measured by cysteine-directed (~40%) and protein-directed (>90%) ABPP, we assessed BcpAc reactivity with HCT116 cells stably expressing recombinant FLAG epitope-tagged TRMT112 (HCT116_TRMT112__–FLAG_). Despite strong expression of recombinant TRMT112 as determined by anti-FLAG immunoblotting, we observed negligible signals for reactivity of this protein with the preferred stereoprobe FWG-3B by gel-ABPP (Fig. 2b). Following anti-FLAG immunoprecipitation (IP), the stereoselective engagement of TRMT112 by FWG-3B and the stereoselective blockade of this engagement by FWG-1B could be observed, indicating that a portion of the recombinant protein retained the reactivity profile observed for endogenous TRMT112 (Fig. 2b). Consistent with a small fraction of recombinant TRMT112 existing in a ligandable state, anti-FLAG IP–MS experiments failed to detect substantial reductions in the unmodified C100-containing tryptic peptide in FWG-1B-treated cells (Extended Data Fig. 5e). We also attempted to express and characterize C100A and C100S mutants of TRMT112 but these variants were produced at very low levels in HCT116 cells (Extended Data Fig. 5f).

Our ABPP data suggested that the BcpAc stereoprobes may react with a specific proteoform of TRMT112. We next set out to identify this proteoform.

#### BcpAc stereoprobes engage a TRMT112:METTL5 complexoform

**Extended Data Fig. 6:**
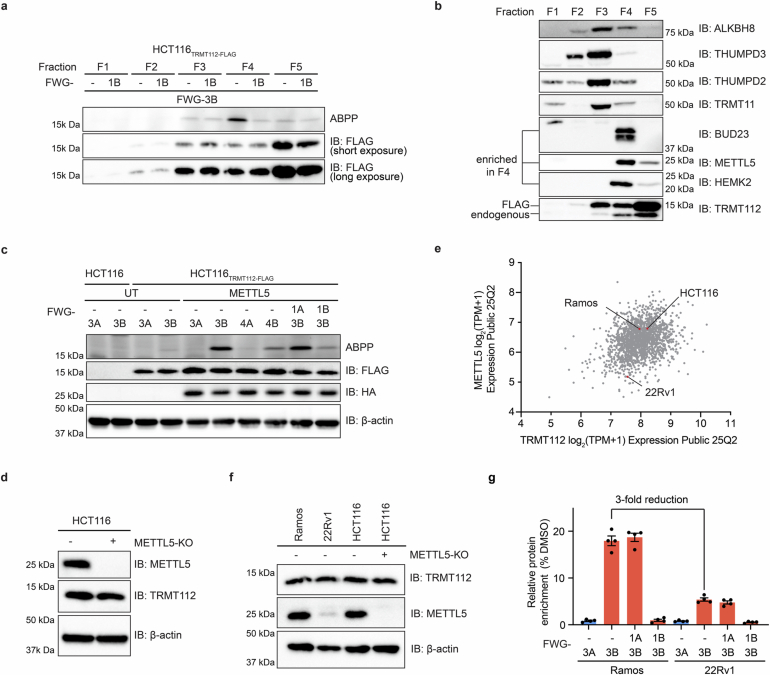
BcpAc stereoprobes engage a TRMT112:METTL5 complexoform in cells. **a**, Gel-ABPP data demonstrating blockade of reactivity of FWG-3B (10 µM, 1 h) with TRMT112 in SEC fraction 4 by pre-treatment of HCT116_TRMT112-FLAG_ cells with FWG-1B (50 µM, 3 h) (see workflow in Fig. 2c). **b**, Western blots showing SEC elution profiles of TRMT112-associated MTs and TRMT112 from SEC-fractionated lysates of HCT116_TRMT112-FLAG_ cells. **c**, Gel-ABPP data demonstrating blockade of the FWG-3B-TRMT112 interaction in METTL5-co-expressing HCT116_TRMT112-FLAG_ cells by FWG-1B, but not FWG-1A. HCT116_TRMT112-FLAG_ cells were transiently transfected with a cDNA for HA-METTL5 and pre-treated with FWG-1A or 1B (50 μM, 3 h), followed by FWG-3A, 3B, 4A, and 4B (10 μM, 1 h), and analyzed by gel-ABPP (as described in Fig. 1b). **d**, Western blot showing similar expression of TRMT112 in parental vs METTL5-KO HCT116 cells. **e**, Scatter-plot showing the expression of TRMT112 (*x*-axis) and METTL5 (*y*-axis) across different human cancer cell lines as measured by RNA-seq. Data are from https://depmap.org/portal/. TPM = transcripts per million. **f**, Western blot showing expression of METTL5 and TRMT112 proteins in Ramos, 22Rv1 and HCT116 cells. For **a-c**, **d** and **f**, data are from a single experiment representative of two independent experiments; IB = immunoblot; UT = untransfected. **g**, Protein-directed ABPP data for TRMT112 from Ramos or 22Rv1 cells pre-treated with FWG-1A and FWG-1B (50 μM, 3 h) or DMSO, followed by alkynes FWG-3A and FWG-3B (10 μM, 1 h). Data are average values ± s.e.m. for four independent biological experiments. Source data

The anti-FLAG IP–MS experiments described above also identified substantial coenrichment of several previously reported MT interaction partners of TRMT112 (ref. ^37^): THUMPD2, TRMT11, HEMK2, THUMPD3, BUD23, METTL5 and ALKBH8 (Extended Data Fig. 5g); FWG-1B did not alter these interactions (Extended Data Fig. 5g). Considering that TRMT112 participates in such a diverse array of MT complexes in cells, we contemplated whether the BcpAcs might preferentially react with one of these TRMT112:MT complexoforms. We designed a workflow to test this hypothesis wherein HCT116_TRMT112__–FLAG_ cells were treated with FWG-3A or FWG-3B, lysed and then fractionated by size-exclusion chromatography (SEC) (Fig. 2c). Each SEC fraction was then subject to anti-FLAG IP, CuAAC conjugation of an azide-rhodamine tag and analysis by gel-ABPP. This experiment revealed that FWG-3B predominantly reacted with the proteoform of TRMT112–FLAG migrating in SEC fraction 4, whereas the vast majority of the TRMT112–FLAG protein, as determined by anti-FLAG immunoblotting, was found in fraction 5 with lower quantities spread across fractions 2–4 (Fig. 2d). The reaction of FWG-3B with TRMT112 in fraction 4 was stereoselective (Fig. 2d) and blocked by pretreatment of cells with FWG-1B (Extended Data Fig. 6a), matching the properties of endogenous TRMT112 characterized in Ramos cells.

Additional immunoblotting experiments of the SEC fractions revealed that three TRMT112-interacting MTs (BUD23, METTL5 and HEMK2) were enriched in fraction 4, with other MT partners being found in higher-molecular-weight fractions (Extended Data Fig. 6b). We then transiently coexpressed HA-tagged BUD23, METTL5 and HEMK2 in HCT116_TRMT112__–FLAG_ cells and treated these cells with FWG-3A or FWG-3B followed by gel-ABPP analysis. This experiment revealed that FWG-3B showed enhanced reactivity with TRMT112 exclusively in cells coexpressing the RNA MT METTL5 (Fig. 2e). The FWG-3B–TRMT112 interaction in METTL5-coexpressing cells was stereoselective (Fig. 2e and Extended Data Fig. 6c) and blocked by pretreatment with FWG-1B (Extended Data Fig. 6c).

To determine whether METTL5 was also required for BcpAc reactivity with endogenous TRMT112, we generated *METTL5*-knockout (KO) HCT116 cells using CRISPR–Cas9 methods (Extended Data Fig. 6d). Protein-directed ABPP experiments revealed a complete loss of stereoselective enrichment of TRMT112 by FWG-3B in *METTL5*-KO cells compared with parental HCT116 cells, while other FWG-3B-enriched proteins were generally unaffected (Fig. 2f and Supplementary Data 1). Additionally, the *METTL5*-KO cells maintained similar expression levels of TRMT112 compared with parental HCT116 cells as determined by immunoblotting (Extended Data Fig. 6d). We finally evaluated whether variation in endogenous expression of METTL5 can modulate the fraction of stereoprobe engagement of TRMT112 in cells. Public RNA sequencing (RNA-seq) data^45^ identified the prostate cancer cell line 22Rv1 as showing substantially reduced expression of METTL5 compared with Ramos cells, whereas both cell lines expressed similar amounts of TRMT112 (Extended Data Fig. 6e). We verified these RNA-seq profiles at the protein level by immunoblotting for METTL5 and TRMT112 (Extended Data Fig. 6f). We then performed protein-directed ABPP experiments, which revealed a threefold greater enrichment of TRMT112 by FWG-3B in Ramos cells compared with 22Rv1 cells (Extended Data Fig. 6g). These results support that the expression level of METTL5 can regulate the fraction of TRMT112 that is engaged by BcpAc stereoprobes.

Taken together, our results support that BcpAcs stereoprobes react with TRMT112 exclusively when this pleiotropic adaptor is bound to METTL5. We next set out to further characterize the structure–activity relationship and mechanism of this complexoform-restricted liganding event.

### Optimization of BcpAc ligands for the TRMT112:METTL5 complex

**Fig. 3:**
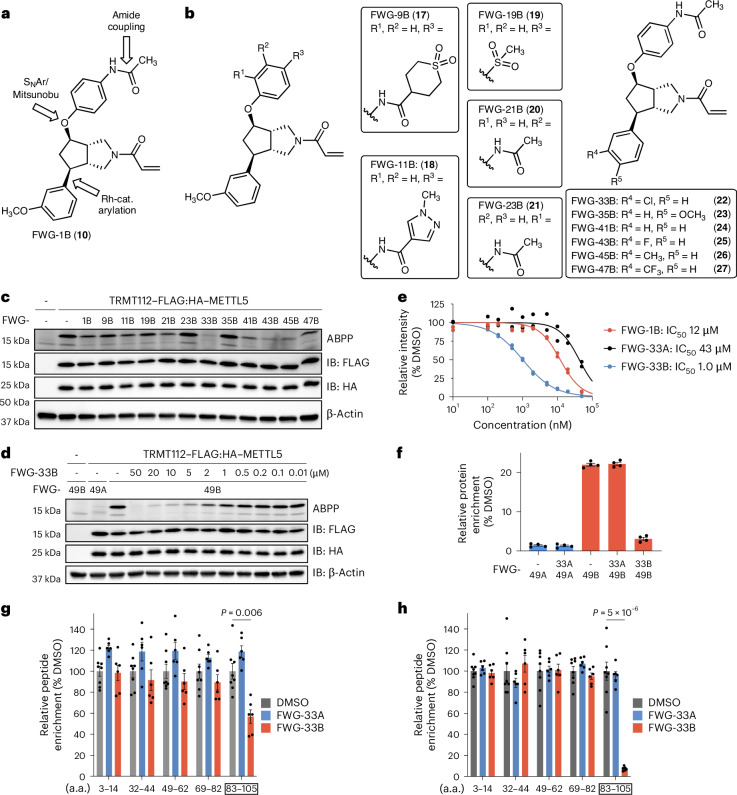
Optimization of BcpAc stereoprobes targeting the TRMT112:METTL5 complex. **a**, Diversification points for BcpAc FWG-1B. Rh-cat., rhodium-catalyzed. **b**, Structures of BcpAc analogs tested for interactions with the TRMT112:METTL5 complex. **c**, Gel-ABPP data showing engagement of TRMT112 by BcpAc analogs in comparison to original ligand FWG-1B. HCT116_TRMT112__–FLAG:HA–METTL5_ cells were pretreated with the indicated BcpAc analogs (20 μM, 3 h) followed by FWG-3B (10 μM, 1 h) and gel-ABPP analysis. Data are from a single experiment representative of two independent experiments. **d**, Gel-ABPP data showing concentration-dependent blockade of the FWG-49B–TRMT112 interaction by FWG-33B. HCT116_TRMT112__–FLAG:HA–METTL5_ cells were pretreated with the indicated concentrations of FWG-33B for 3 h followed by FWG-49A and FWG-49B (2 μM, 1 h) and gel-ABPP analysis. Data are from a single experiment representative of two independent experiments. **e**, Quantification of gel-ABPP data for engagement of TRMT112 by FWG-1B, FWG-33A and FWG-33B in HCT116_TRMT112__–FLAG:HA–METTL5_ cells (Fig. 3d and Extended Data Fig. 7a,d). Data are from two independent biological experiments. **f**, Bar graph showing protein-directed ABPP for endogenous TRMT112 from Ramos cells pretreated with DMSO or FWG-33A or FWG-33B (5 μM, 3 h) followed by FWG-49A and FWG-49B (2 μM, 1 h) and protein-directed ABPP analysis. Data are average values ± s.e.m. for four independent biological experiments. **g**,**h**, Quantification of engagement of TRMT112 C100 by FWG-33B (5 µM, 4 h) as measured by monitoring the C100-containing tryptic peptide in anti-FLAG (**g**) or anti-HA (**h**) IP–MS experiments from HCT116_TRMT112__–FLAG:HA–METTL5_ cells. a.a., amino acids. Data are average values ± s.e.m. normalized to DMSO for six independent biological experiments, with the exception of the DMSO group with eight independent biological replicates. Statistical significance between the DMSO and the FWG-33B-treated peptides was assessed using two-sided, unpaired-multiple *t*-tests (Holm–Šídák approach to multiple comparisons; reported *P* values are adjusted *P* values). Source data

**Extended Data Fig. 7:**
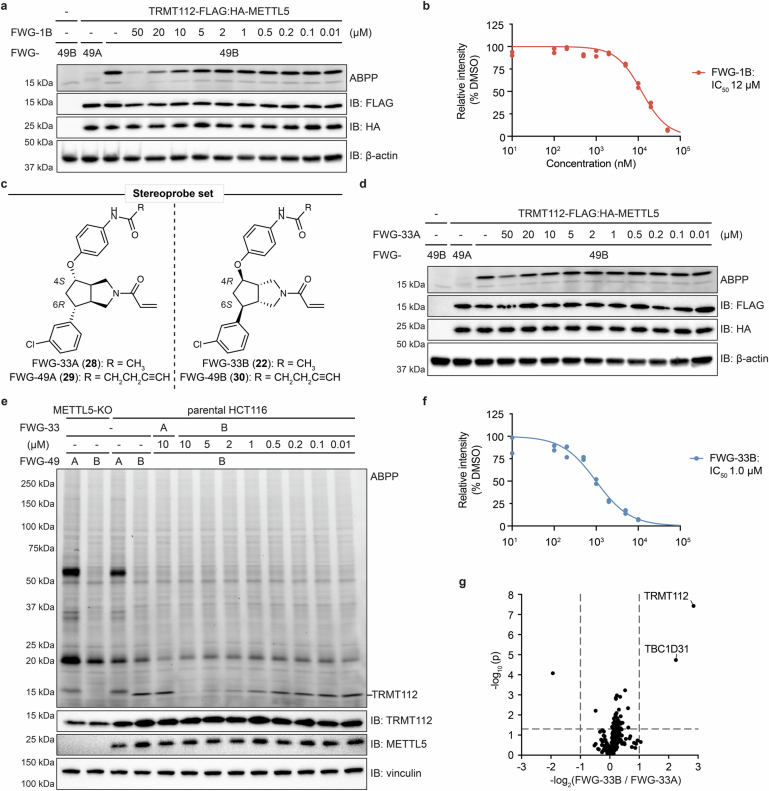
Optimization of BcpAc stereoprobes targeting the TRMT112:METTL5 complex. **a**, Gel-ABPP data showing concentration-dependent blockade of the FWG-49B-TRMT112 interaction by FWG-1B. HCT116_TRMT112-FLAG:HA-METTL5_ cells were pre-treated with the indicated concentrations of FWG-1B for 3 h, followed by FWG-49A and 49B (2 μM, 1 h), and gel-ABPP analysis. Data are from a single experiment representative of two independent experiments. **b**, Quantification of gel-ABPP data from panel **a** for engagement of TRMT112 by FWG-1B in HCT116_TRMT112-FLAG:HA-METTL5_ cells. Data are from two independent biological experiments. **c**, Structures of non-alkyne (FWG-33A (**28**), FWG-33B (**22**)) and alkyne (FWG-49A (**29**), FWG-33B (**30**)) BcpAc stereoprobes. **d**, Gel-ABPP data showing concentration-dependent blockade of the FWG-49B-TRMT112 interaction by FWG-33A. HCT116_TRMT112-FLAG:HA-METTL5_ cells were pre-treated with the indicated concentrations of FWG-33A for 3 h, followed by FWG-49A and 49B (2 μM, 1 h), and gel-ABPP analysis. Data are from a single experiment representative of two independent experiments. **e**, Gel-ABPP data showing METTL5-dependent stereoselective liganding of TRMT112. Parental HCT116 cells or METTL5-KO cells were treated with FWG-33A (10 μM, 3 h) or 33B (10 to 0.01 μM, 3 h), followed by FWG-49A and 49B (2 μM, 1 h), and analyzed by gel-ABPP (as described in Fig. 1b). Data are from a single experiment representative of two independent experiments. For **a**, **d**, **e**, IB = immunoblot. **f**, Quantification of gel-ABPP data from panel **e** for engagement of endogenous TRMT112 by FWG-33B in parental HCT116 cells. Data are from two independent biological experiments. **g**, Volcano plot showing protein-directed ABPP for blockade of FWG-49B enrichment of proteins by FWG-33B versus FWG-33A (same protein-directed ABPP experiment as shown in Fig. 3f). Data are average values for four independent biological experiments. Statistical significance was assessed using Welch two sample, two-sided *t*-tests. Source data

While our initial BcpAc ligand FWG-1B engaged TRMT112 in cells with high stereoselectivity, this interaction showed modest potency as determined by gel-ABPP (half-maximal inhibitory concentration (IC_50_) value = 12 µM; Extended Data Fig 7a,b). We, therefore, performed an initial structure–activity analysis to identify higher-potency ligands for the TRMT112:METTL5 complex. We selected three positions on the Bcp scaffold that could be diversified using robust chemical transformations, including amide coupling, S_N_Ar/Mitsunobu reaction and rhodium-catalyzed arylation (Fig. 3a). Analogs at the C4 substitution produced only subtle differences in potency as determined by gel-ABPP experiments performed in HCT116_TRMT112__–FLAG:HA–METTL5_ cells, with the exception of FWG-23B, which featured the *N*-(2-phenyl)acetamide substitution and exhibited a marked decrease in potency (Fig. 3b,c). In contrast, modifications at the C6 position resulted in much more pronounced differences in reactivity with TRMT112, with the 3-chlorophenyl analog FWG-33B showing the greatest apparent improvement in potency (Fig. 3b,c).

We next generated alkyne analogs of FWG-33B and its enantiomer FWG-33A—FWG-49B and FWG-49A (Extended Data Fig. 7c)—and used these compounds as target engagement probes in subsequent gel-ABPP experiments measuring the concentration-dependent reactivity of FWG-1B, FWG-33A and FWG-33B with TRMT112 in HCT116_TRMT112__–FLAG:HA–METTL5_ cells (Fig. 3d,e and Extended Data Fig. 7a,d). These experiments revealed a ~10-fold improvement in potency for FWG-33B compared with FWG-1B (IC_50_ values: FWG-1B, 12 μΜ; FWG-33B, 1.0 μΜ) with maintenance of stereoselective reactivity (FWG-33A > 40 μΜ). FWG-49B also appeared to share this increase in potency, as this alkyne stereoprobe proved capable of visualizing endogenous TRMT112 at 2 µM test concentrations in HCT116 cells (Extended Data Fig. 7e,f). We next evaluated the proteome-wide reactivity of FWG-33B and alkyne analog FWG-49B by protein-directed ABPP in Ramos cells. These experiments confirmed robust stereoselective enrichment of TRMT112 in cells treated with FWG-49B versus FWG-49A and the near-complete (~90%) blockade of this enrichment by pretreatment with FWG-33B (5 μM, 3 h) (Fig. 3f). In contrast, pretreatment with the enantiomer FWG-33A did not perturb enrichment of TRMT112 by FWG-49B (Fig. 3f). One additional stereoselective target of FWG-33B was identified in this experiment—TBC1D31 (Extended Data Fig. 7g and Supplementary Data 1), which is an adaptor protein involved in cilium biogenesis^46^.

We next assessed the fraction of total and METTL5-complexed TRMT112 that reacted with FWG-33B in HCT116_TRMT112__–FLAG:HA–METTL5_ cells as measured by IP–MS using anti-FLAG and anti-HA antibodies, respectively. We surmised that, in these experiments, signals for the tryptic peptide containing C100 of TRMT112 (amino acids 83–105) would provide an estimate of FWG-33B engagement of total (anti-FLAG) versus METTL5-complexed (anti-HA) TRMT112. The anti-FLAG IP–MS revealed a ~40% reduction in unmodified peptide (amino acids 83–105) in FWG-33B-treated cells (Fig. 3g), suggesting that, in cells recombinantly expressing both TRMT112 and METTL5, ~40% of the total TRMT112 was engaged by FWG-33B. In contrast, the signals for the unmodified peptide (amino acids 83–105) were decreased >90% in anti-HA IP–MS experiments from these same cells (Fig. 3h), supporting that the fraction of TRMT112 interacting with METTL5 was fully engaged by FWG-33B in cells.

### Crystal structure of TRMT112:METTL5 bound to FWG-33B

**Fig. 4:**
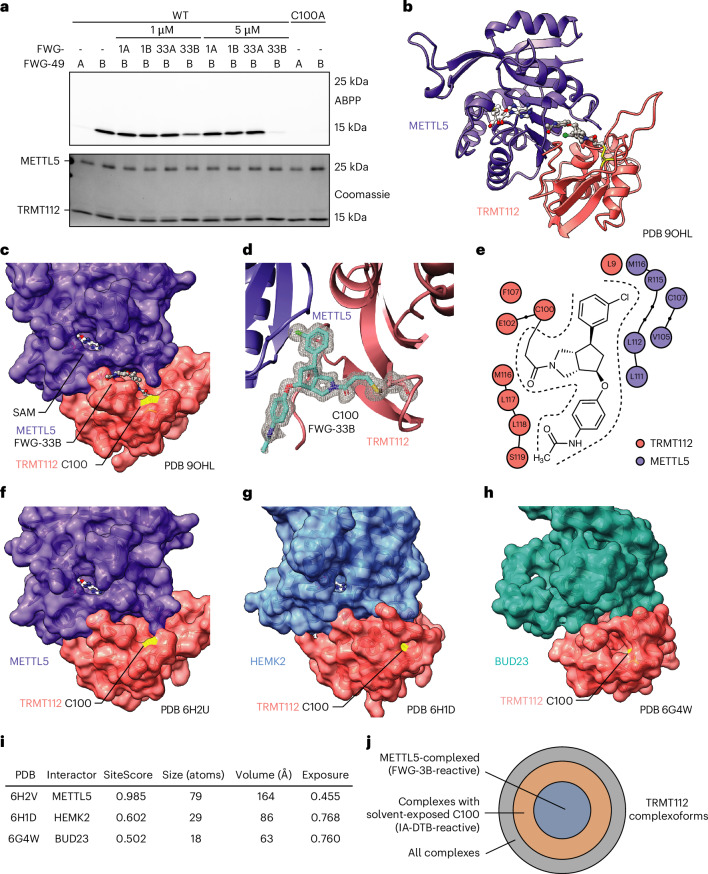
Crystal structure of a FWG-33B-modified TRMT112:METTL5 complex. **a**, Gel-ABPP data showing stereoselective and site-specific liganding of purified WT-TRMT112:WT-His–METTL5 with FWG-49B. Purified WT-TRMT112:WT-His–METTL5 complex (1 μM) was pretreated with FWG-1A, FWG-1B, FWG-33A or FWG-33B (1 or 5 μM, 1 h), followed by FWG-49A or FWG-49B (1 μM, 1 h), and analyzed by gel-ABPP (as described in Fig. 1b). Data are from a single experiment representative of two independent experiments. **b**, Cocrystal structure of the TRMT112–METTL5 complex with FWG-33B and SAM (PDB 9OHL, 1.3 Å). **c**, Surface area representation of the TRMT112–METTL5 complex showing location of FWG-33B and SAM (C100 shown in yellow). **d**, *F*_O_ − *F*_C_ omit map contoured at 3*σ* showing clear density for FWG-33B continuous with TRMT112 C100. **e**, Protein–ligand interaction diagram highlighting residues in TRMT112 and METTL5 that interact with FWG-33B. Amino acids within 4 Å of FWG-33B are shown (analyzed with Schrödinger Maestro). Small black dots represent amino acids in the chain that are not within 4 Å of FWG-33B. **f**–**h**, Crystal structures of an apo TRMT112:METTL5 complex (**f**; PDB 6H2U), and TRMT112:HEMK2 complex (**g**; PDB 6H1D) and a cryo-electron microscopy structure of the TRMT112:BUD23 complex (**h**; PDB 6G4W). **i**, Site mapping with Schrödinger Maestro near TRMT112 C100 reveals large differences in apparent solvent accessibility for the TRMT112:METTL5 complex compared with the TRMT112:HEMK2 and TRMT112:BUD23 complexes. **j**, Concentric circle diagram showing TRMT112 complexoforms as relates to their potential reactivity with BcpAcs (for example, FWG-3B) and/or IA-DTB. Source data

We expressed wild-type (WT)-TRMT112:METTL5 and TRMT112-C100A:METTL5 complexes in *Escherichia* *coli* (BL21) and purified these complexes by sequential metal affinity, anion exchange and SEC. FWG-49B reacted stereoselectively with the purified WT-TRMT112:METTL5 complex but not the TRMT112-C100A:METTL5 complex and pretreatment with FWG-33B but not FWG-33A (5 µM each, 1 h) blocked FWG-49B reactivity with WT-TRMT112 (Fig. 4a).

**Extended Data Fig. 8:**
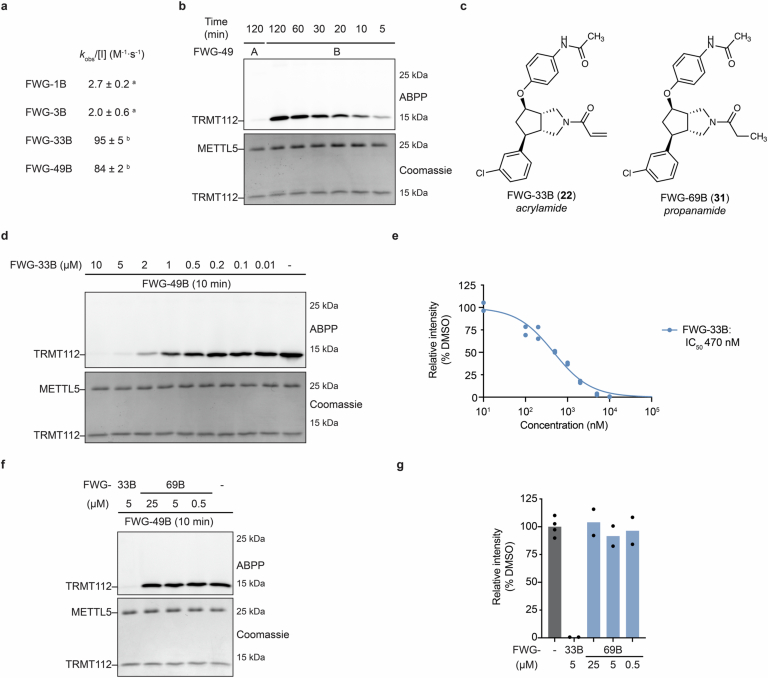
Further characterization of TRMT112:METTL5 ligands. **a**, *k*_obs_/[I] values for stereoprobe reactivity with TRMT112. Purified WT-TRMT112:His-METTL5 complex (0.5 μM) was treated with FWG-1B, 3B, 33B, or 49B (10 μΜ) for 60 (^a^) or 10 (^b^) min, followed by addition of formic acid (0.5%). The modification of TRMT112 with stereoprobes was quantified by intact MS and used to calculate *k*_obs_/[I] assuming pseudo-first order kinetics. Data are average values ± s.e.m. for three independent experiments (see Supplementary Fig. 2 and Supplementary Table 1). **b**. Gel-ABPP data showing time-dependent reactivity of purified WT-TRMT112:His-METTL5 complex with FWG-49B. The purified TRMT112:METTL5 complex (1 μM) was treated with FWG-49A or 49B (0.5 μM) for the indicated times and analyzed by gel-ABPP (as described in Fig. 1b). Data are from a single experiment representative of two independent experiments. **c**, Structures of acrylamide FWG-33B (**22**) and non-electrophilic propanamide analogue FWG-69B (**31**). **d**, Gel-ABPP data showing concentration-dependent blockade of the FWG-49B-TRMT112 interaction by FWG-33B with purified TRMT112: METTL5 complex. The TRMT112:METTL5 complex (1 μM) was pre-treated with the indicated concentrations of FWG-33B (1 h), followed by FWG-49B (0.5 μM, 10 min), and analyzed by gel-ABPP (as described in Fig. 1b). Data are from a single experiment representative of two independent experiments. **e**, Quantification of gel-ABPP data from panel **d**. Data are from two independent experiments. **f**, Gel-ABPP data showing that FWG-69B across a test concentration range of 0.5 – 25 µM does not block the FWG-49B-TRMT112 interaction. Purified TRMT112:METTL5 complex (1 μM) was pre-treated with the indicated concentrations of FWG-69B (25, 5, or 0.5 μΜ, 1 h) or FWG-33B (5 µM, 1 h), followed by FWG-49B (0.5 μM, 10 min), and analyzed by gel-ABPP (as described in Fig. 1b). Data are from a single experiment representative of two independent experiments. **g**, Quantification of gel-ABPP data from panel **f**. Data are average values for two independent experiments, with the exception of the DMSO group, which has four independent replicates. Source data

We further confirmed BcpAc reactivity with the purified WT-TRMT112:METTL5 complex by intact MS analysis and used this method to measure a *k*_obs_/[*I*] value of 95 ± 5 M^−1^ s^−1^ for FWG-33B, where *I* is the inhibitor concentration (Extended Data Fig. 8a and Supplementary Fig. 2). This value was >30-fold higher than the *k*_obs_/[*I*] values of original methoxy compounds FWG-1B and FWG-3B (Extended Data Fig. 8a), mirroring the increase in potency observed for FWG-33B in cells (Fig. 3e). We additionally established a kinetically controlled ABPP reaction between FWG-49B and the purified WT-TRMT112:METTL5 complex (Extended Data Fig. 8b). Under these conditions, we confirmed engagement of the TRMT112:METTL5 complex by FWG-33B (IC_50_ value, 0.47 µM) but were unable to measure reversible binding of a nonelectrophilic propanamide analog of FWG-33B (FWG-69B; Extended Data Fig. 8c) across a test concentration range of 0.5–25 µM (Extended Data Fig. 8d–g). We interpret these data to indicate that the potency of FWG-33B as a TRMT112:METTL5 ligand is mostly driven by covalent reactivity.

**Extended Data Fig. 9:**
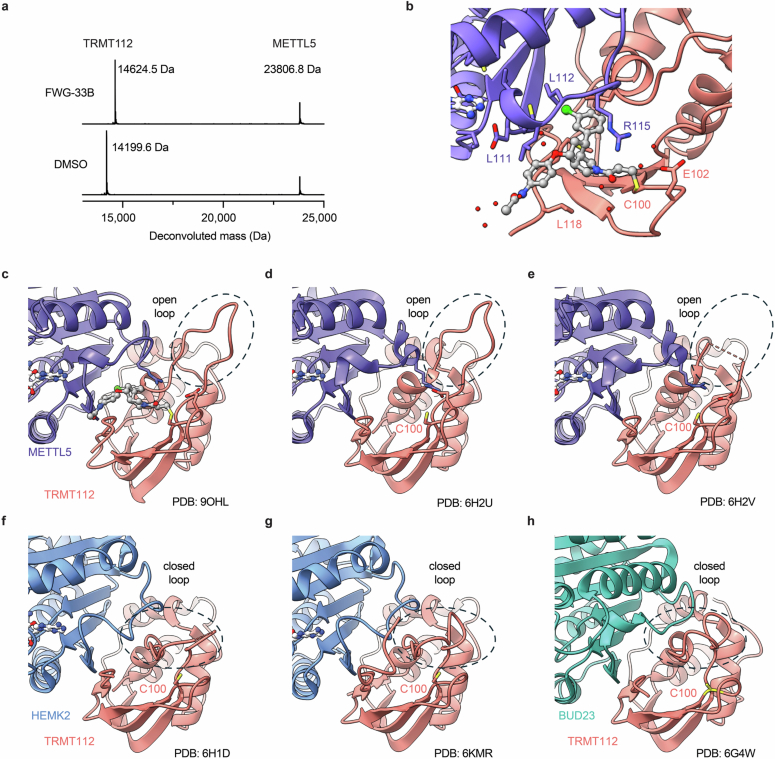
Crystal structure of an FWG-33B-modified TRMT112:METTL5 complex. **a**, Intact MS showing complete modification of TRMT112 by FWG-33B. Purified wild-type (WT)-TRMT112:WT-METTL5 complex (20 μM) treated with FWG-33B (100 μM, 30 min) in presence of S-adenosylmethionine (SAM) (1 mM), and analyzed by Q-TOF MS. Data are from a single experiment. Da. = Dalton. **b**, Key residues in the FWG-33B binding site. Blue, METTL5; salmon, TRMT112. **c-h**, TRMT112 structures in TRMT112:METTL5 (**c**-**e**), TRMT112:HEMK2 (**f**-**g**) and TRMT112:BUD23 (**h**) showing mostly good alignment. A dynamic loop (black circle, TRMT112: a.a. L9-F21) in proximity to C100 is highlighted to show its open conformation in apo TRMT112:METTL5 structures (PDB 6H2U (**d**) and in PDB 6H2V (**e**)) and closed conformation in other TRMT112:MT structures (TRMT112:HEMK2: PDB 6H1D (**f**) and PDB 6KMR (**g**); TRMT112:BUD23: PDB 6G4W (**h**)).

We next identified conditions for the formation of a single, quantitative adduct of FWG-33B with TRMT112:METTL5 by intact MS (Extended Data Fig. 9a), which facilitated determination of the structure of the FWG-33B-liganded TRMT112:METTL5 complex in the presence of the SAM cofactor by X-ray crystallography. This 1.3-Å crystal structure (PDB 9OHL; Supplementary Table 2) revealed that FWG-33B binds in a well-formed pocket residing at the interface of TRMT112 and METTL5 that is fully distinct from the SAM-binding pocket of METTL5 (~22.5 Å between SAM (CH_3_) and FWG-33B (C6a)) (Fig. 4b,c). Clear electron density confirmed a covalent adduct between the acrylamide of FWG-33B and C100 of TRMT112 (Fig. 4d). FWG-33B interacts extensively with both TRMT112 (total surface area of interaction: 301 Å^2^) and METTL5 (total surface area of interaction: 235 Å^2^) (Fig. 4e). A tight fit is observed in this composite pocket for the 3-chlorophenyl group (C6) of FWG-33B, which interacts with hydrophobic residues V105, C107, L112, R115 and M116 of METTL5, as well as L9 and M116 of TRMT112 (Fig. 4e and Extended Data Fig. 9b). This series of interactions aligns with the steep structure–activity relationship displayed by various C6 analogs (Fig. 3b,c). In contrast, the C4 appendage extends away from the binding pocket into a solvent-exposed region (Extended Data Fig. 9b), which is consistent with the limited impact of C4 modifications on BcpAc engagement of the TRMT112:METTL5 complex.

A comparison of the structure of the FWG-33B-liganded TRMT112:METTL5 complex to previously reported apo structures of TRMT112:METTL5 (ref. ^38^) (Fig. 4f), TRMT112:HEMK2 (Fig. 4g) and TRMT112:BUD23 (Fig. 4h) helped to explain the basis for exclusive reactivity of BcpAcs with TRMT112 when bound to METTL5, as neither the TRMT112:HEMK2 (ref. ^47^) nor TRMT112:BUD23 (ref. ^48^) complex displayed evidence of a solvent-accessible pocket in proximity to C100 of TRMT112. These differences appear to be at least partly explained by the location of a dynamic loop in close proximity to C100 (L9–F21 of TRMT112), which is open in FWG-33B-liganded and apo TRMT112:METTL5 structures (Extended Data Fig. 9c–e) but closed in other TRMT112:MT structures^47–49^ (Extended Data Fig. 9f–h), although not fully resolved in all structures (Extended Data Fig. 9f).

Site mapping performed in Schrödinger Maestro supported major differences in the apparent solvent accessibility near TRMT112 C100, with only the TRMT112:METTL5 complex having a well-defined predicted pocket (164 Å^3^; Fig. 4i). These structural data help to rationalize the complexoform-restricted liganding of TRMT112 when bound to METTL5 and may also explain the discordance between our cysteine-directed and protein-directed ABPP data for C100 of TRMT112 (Fig. 2a). It is possible, for instance, that the IA-DTB probe used in cysteine-directed ABPP experiments can react with C100 in multiple TRMT112 complexoforms but the alkynylated BcpAcs used in protein-directed ABPP experiments exclusively engage TRMT112 when bound to METTL5. The resulting profiles would then lead to maximal and submaximal engagement estimates for BcpAc competitors in protein-directed and cysteine-directed ABPP experiments, respectively, with the latter matching the fraction of total IA-DTB-reactive TRMT112 C100 residing in TRMT112:METTL5 complexes (Fig. 4j).

### TRMT112 ligands allosterically potentiate METTL5 activity

**Fig. 5:**
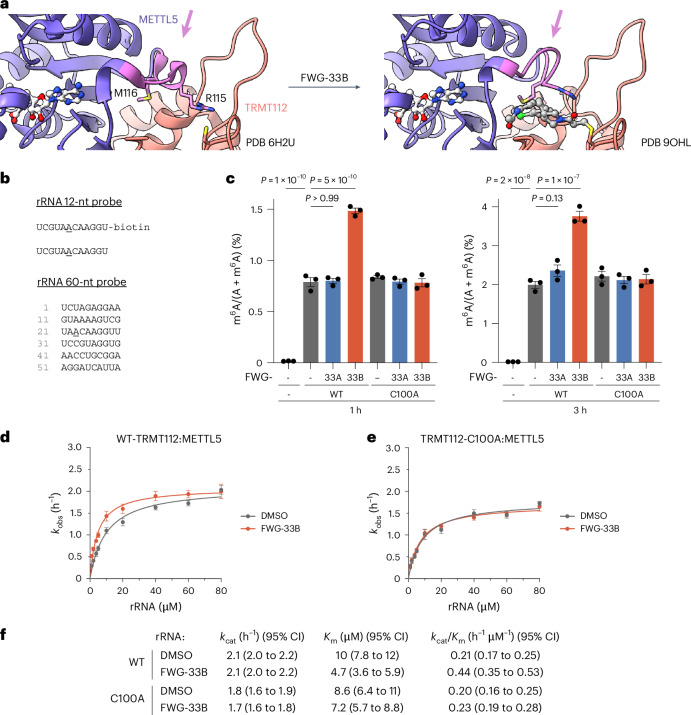
FWG-33B enhances the MT activity of METTL5. **a**, FWG-33B induces allosteric changes in the TRMT112:METTL5 structure, including movement of a flexible loop (colored in magenta, amino acids L111–S117 in METTL5) and unwinding of single-turn helix in proximity to the SAM-binding site. **b**, Model RNA substrates (12 or 60 nt) containing the A_1832_ motif of the 18S ribosomal RNA (A_1832_ underlined). **c**, Effects of FWG-33B on the catalytic activity of WT-TRMT112:His–METTL5 and TRMT112-C100A:His–METTL5 complexes using a biotinylated 12-nt substrate. TRMT112:His–METTL5 complexes (20 μM) were treated with FWG-33A or FWG-33B (40 μM, 30 min) or DMSO in the presence of SAM (1 mM). The samples were then diluted 20-fold into the METTL5 activity assay buffer (10 μM biotinylated RNA, 1 mM SAM, 5 mM MgCl_2_, 50 mM NaCl, 50 mM Tris and 1 mM DTT) for 1 or 3 h (37 °C), followed by enrichment using streptavidin beads and digestion for the RNA substrates into single nucleosides and targeted (triple quadrupole) LC–MS analysis. Data are from a single experiment containing three technical replicates and representative of two independent experiments. Data are average values ± s.e.m. Statistical significance was assessed using a one-way ANOVA. **d**,**e**, Kinetic analysis of WT-TRMT112:His–METTL5 (**d**) and TRMT112-C100A:His–METTL5 (**e**) activity with variable concentrations of a 12-nt rRNA substrate and a saturating concentration of SAM. TRMT112:His–METTL5 complexes (20 μM) were treated with FWG-33B (40 μM, 30 min) or DMSO. The samples were then diluted 1000-fold into the METTL5 activity assay buffer (0.2 μM enzyme, 1–80 μM RNA, 40 μM SAM, 50 mM NaCl, 20 mM Tris and 1 mM DTT) for 20 min (room temperature), followed by the addition of TFA (0.1%). SAH was quantified by the MTase-Glo assay. Data are average values ± s.e.m. from two independent experiments each containing three technical replicates. **f**, Determination of Michaelis–Menten kinetic parameters (*k*_cat_, *K*_m_ and *k*_cat_/*K*_m_) from **d** and **e**. The 95% CIs are shown in brackets. Source data

A more detailed comparison of the structures of the apo and FWG-33B-liganded TRMT112:METTL5 complexes revealed a marked rearrangement in a flexible loop of METTL5 (L111–S117) near the SAM-binding site (Fig. 5a). In the unliganded structures, S113–S117 forms a single-turn helix that unfolds upon FWG-33B binding to place the ε carbon of METTL5 M116 and the β and γ carbons of METTL5 R115 against the chlorophenyl moiety. The extensive interactions between L111–S117 and FWG-33B, along with the hydrogen-bond-mediated coordination of the adenine base of the SAM cofactor by the proximal residues D108 and V109, suggested that FWG-33B reactivity with TRMT112 C100 might allosterically modulate the catalytic activity of METTL5.

**Extended Data Fig. 10:**
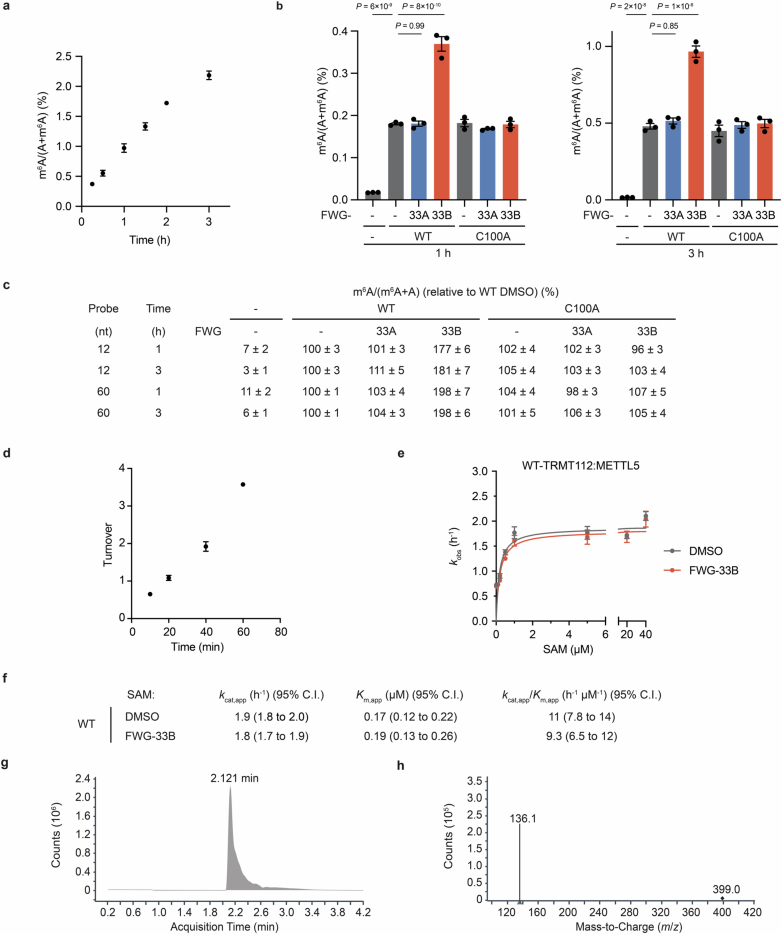
FWG-33B enhances the MT activity of METTL5. **a**, Time-dependent activity for the wild-type (WT)-TRMT112:His-METTL5 complex using a biotinylated 12 nt RNA substrate. Data are average values ± s.e.m. from three independent experiments. **b**, Effects of FWG-33B or FWG-33A (40 µM, 30 min) on the catalytic activity of WT-TRMT112:His-METTL5 and C100A-TRMT112:His-METTL5 complexes (20 µM) using a 60 nt substrate (10 µM). Reactions run in presence of SAM (1 mM) and as described in the Methods. Data are average values ± s.e.m. from a single experiment containing three technical replicates representative of two independent experiments. Statistical significance was assessed using one-way analysis of variance (ANOVA). **c**, Table summarizing METTL5 activity data reported as average values ± s.e.m. for two experiments with each three technical replicates. For **a-c**, A = adenosine; m6A = *N*^6^-methyladenosine. **d**, Time-dependent activity of the WT-TRMT112:His-METTL5 complex using a 12 nt RNA substrate. Reactions were performed as described in Fig. 5d. Data are average values ± s.e.m. from three independent experiments. **e**, Kinetic analysis for WT-TRMT112:His-METTL5 methyltransferase reactions with a 12 nt rRNA substrate under saturating RNA concentrations. The TRMT112:METTL5 complex (20 μM) were treated with FWG-33B (40 μM) or DMSO for 30 min, and samples were analyzed as described in Fig. 5d. Data are average values ± s.e.m. from three independent experiments containing each two-three technical replicates. **f**, Determination of apparent Michaelis–Menten kinetic parameters (*k*_cat, app_, *K*_m, app_, and *k*_cat, app_ /*K*_m, app_) from panel **e**. 95% confidence intervals (C.I.) are shown in brackets. Data was fitted excluding the no SAM concentration point. **g** and **h**, Multiple reaction monitoring (MRM) quantification of SAM in purified WT-TRMT112:METTL5 complex following methanol denaturation using an *m/*z (399.0 → 136.1) transition. Representative total ion chromatogram for the [M]^+^ ion (**g**) and spectrum of the MRM transition (**h**) are shown. Data are from a single experiment representative of two independent experiments. Source data

The TRMT112:METTL5 complex catalyzes the *N*^6^-methyladenosine (m^6^A) modification at A_1832_ of the 18S small ribosomal subunit^38,50,51^. This modification occurs near the ribosomal decoding center, where it influences translation efficiency and specificity^38,50,51^. We tested the impact of FWG-33B on METTL5 activity using single-stranded RNA substrates containing the UAACA motif derived from the 18S ribosomal RNA where the underlined A corresponds to A_1832_ (refs. ^51–53^; Fig. 5b). The purified WT-TRMT112 or TRMT112-C100A:METTL5 complexes (20 μΜ protein) were treated with FWG-33A or FWG-33B (40 μΜ) or DMSO control for 30 min, after which the proteins were diluted 20-fold into the substrate assay buffer containing RNA substrate (10 µM) and SAM (1 µM); after 1 or 3 h, the methylated RNA product was quantified by measuring the ratio of A and m^6^A by liquid chromatography (LC)–MS^51,52^. The DMSO control experiments confirmed that both WT-TRMT112:METTL5 and TRMT112-C100A:METTL5 complexes exhibited similar time-dependent methylation activities with the short (12 nt) or long (60 nt) substrate (Fig. 5c and Extended Data Fig. 10a–c). The FWG-33B-treated WT-TRMT112:METTL5 complex exhibited a ~2-fold increase in methylation activity with each RNA substrate that was both stereoselective and not observed with the TRMT112-C100A:METTL5 complex (Fig. 5c and Extended Data Fig. 10b,c).

To investigate in more detail how FWG-33B stimulates TRMT112:METTL5 activity, we established a higher-throughput MTase-Glo assay^53^ (Extended Data Fig. 10d) that quantifies the formation of *S*-adenosyl-L-homocysteine (SAH). Purified WT-TRMT112 or TRMT112-C100A:METTL5 complexes (20 μΜ protein) were treated with FWG-33B (40 μΜ) or DMSO control for 30 min, after which the proteins were diluted 100-fold into the substrate assay buffer containing varying concentrations of a 12-nt rRNA oligo substrate and SAM. The reactions were stopped after 20 min and SAH was measured by luminescence^53^. Under saturating concentrations of SAM (Fig. 5d,e), FWG-33B caused a twofold increase in catalytic efficiency (*k*_cat_/*K*_m_) accounted for by a twofold reduction in the *K*_m_ value for the RNA substrate (Fig. 5d,f). As expected, FWG-33B did not alter the *k*_cat_/*K*_m_ value for the TRMT112-C100A:METTL5 mutant (Fig. 5e,f). In contrast, in the presence of saturating concentrations of RNA substrate and varying amounts of SAM, FWG-33B did not substantially affect the kinetic parameters of the WT-TRMT112:METTL5 complex (Extended Data Fig. 10e,f). We should note, however, that the WT-TRMT112:METTL5 complex showed appreciable activity without the addition of SAM (Extended Data Fig. 10e), suggesting that some endogenous SAM copurified with the complex from *E*. *coli*, as has been observed for other MTs, including the TRMT112:HEMK2 complex^49,54^. Consistent with this hypothesis, we detected SAM in the purified WT-TRMT112:METTL5 complex after denaturation with methanol and LC–MS analysis (Extended Data Fig. 10g,h). Acknowledging that the basal level of WT-TRMT112:METTL5 activity observed in the absence of exogenous SAM complicates the determination of absolute kinetic parameters, we refer to the *k*_cat_ and *K*_m_ values under varying concentrations of SAM as apparent values.

Our combined structural and enzyme kinetic data support that covalent binding of FWG-33B to an interface pocket of the TRMT112:METTL5 complex leads to allosteric enhancement of METTL5 catalytic activity by lowering its *K*_m_ value for rRNA substrate, thus providing a chemical tool for studying the potentiation of METTL5 activity in biological systems.

## Discussion

The massive structural and functional diversity conferred by proteoforms presents an exciting opportunity for chemical biology and drug discovery^1,7,55–57^. Small molecules that target specific proteoforms have the potential to impact cell and human biology with a level of precision that exceeds the complete genetic or pharmacological disruption of proteins. While, in select instances, a specific proteoform may be amenable to production (for example, semisynthesis^58^), purification and screening, the general pursuit of proteoform-restricted ligands would benefit from methodologies that operate in native biological systems where a more complete range of post-translational protein states can be assayed. Here, we showed that ABPP offers a compelling strategy for identifying covalent ligands that target a single complexation state (or complexoform^5,6^) of the general MT adaptor TRMT112.

The discordant profiles of TRMT112 in cysteine-directed and protein-directed ABPP datasets (incomplete and complete liganding, respectively) provided the first clue that BcpAc stereoprobes engage this protein in an atypical manner. The extent to which such discrepancies can serve as a general hallmark of proteoform-restricted liganding events will depend on the depth of coverage in cysteine-directed ABPP datasets, as alternative explanations, such as the failure to detect the relevant cysteine for a liganding event mapped exclusively by protein-directed ABPP, remain possible. Nonetheless, we believe that some of the additional experimental approaches deployed in this study may prove generally useful for mapping complexoform-restricted small-molecule interactions. For instance, we envision that integrating ABPP with the proteomic analysis of SEC fractions from cells treated with electrophilic stereoprobes may provide a robust way of identifying liganding events that exclusively occur with rare complexoforms of proteins. Correlating ABPP and protein expression data across different cell lines may offer another method for the systematic discovery of liganding events with rare complexoforms. Combining ABPP with top-down and native protein MS^5,59^, as well as other global measurements of protein modifications (for example, phosphoproteomics^7,60^), should also serve as complementary approaches for relating covalent liganding events to individual proteoforms in cells.

The role of the METTL5-mediated 18S rRNA m^6^A_1832_ modification in regulating protein translation and downstream cellular processes is under active investigation in fields ranging from neuroscience^61,62^ to metabolism^63^ and cancer^63,64^. Deleterious mutations in *METTL5* lead to intellectual disability and microcephaly in humans^65^, pointing to an important role for this enzyme in brain development. Additionally, METTL5 has been implicated in tumorigenesis, including supporting the growth of breast^52^, hepatocellular^63^ and nasopharyngeal^64^ carcinomas. Chemical probes, however, have been lacking for METTL5; therefore, we believe that the allosteric agonists reported herein can offer valuable tools for studying the functions of this enzyme in diverse cellular processes. We should note, however, that the basal level of 18S rRNA m^6^A_1832_ modification is quite high (>98%) in immortalized cells grown under standard cell culture conditions^50,63,66^, which indicates that alternative cell models may be needed to study the impact of METTL5 agonism on ribosome function and protein translation. Some studies suggest that METTL5 may also regulate the methylation of mRNAs^67^ and evaluating the effects of FWG-33B on the global m^6^A profiles of cells^68–70^ may help to establish the full substrate scope of METTL5.

From a translational perspective, METTL5 agonists may be beneficial for neurodegenerative disorders, considering a recent study that points to a role for METTL5 in promoting corticospinal tract sprouting following brain injury^62^. For diseases such as cancer, however, METTL5 inhibitors would appear more relevant. We note, in this regard, that allosteric sites on other proteins, including E3 ligases (for example, KEAP1 (ref. ^71^)) and kinases (for example, BCR-ABL^72^), have been found to support both small-molecule-mediated agonism and antagonism. We, therefore, wonder whether the TRMT112:METTL5 composite pocket may also serve as a future source for METTL5 inhibitors.

Small molecules that induce protein–protein interactions through the formation of ternary complexes have become a major source of chemical probes and drugs^73^. Such induced proximity mechanisms can strengthen dynamic protein–protein interactions or promote novel ones. Our findings highlight an alternative and complementary mechanism for small-molecule regulation of stable protein complexes. By binding at a composite pocket unique to the interface of TRMT112 and METTL5, FWG-33B not only achieves high selectivity for a single MT partner of TRMT112 but also causes allosteric effects that increase the methylation activity of METTL5. This type of ‘proximity-induced’ liganding event was recently observed for allosteric inhibitors of the CCNE1:CDK2 complex, which bind to a composite pocket distal from the CDK2 active site and similarly display complexoform-restricted activity (in this case, over other cyclin:CDK2 complexes, such as CCNE2:CDK2)^74^. We do not yet understand the frequency of allosteric composite pockets across the human proteome but the cases reported to date underscore the importance of considering each complexoform as an independent source.

Looking forward, we believe that additional general insights can be drawn from the overall chemical proteomic profiles of the BcpAcs. These compounds, despite exhibiting lower intrinsic reactivity compared with previously described azetidine and tryptoline acrylamide stereoprobes, engaged several unique proteins in human cells (Table 1). Thus, our results, alongside studies of additional classes of lower-reactivity stereoprobes^21^, emphasize the value of scaffold diversity in the design of electrophilic compound libraries. Toward this end, the catalytic stereoselective synthetic strategy described for the BcpAcs may offer a useful approach for scaffold diversification compared with past routes for stereoprobe construction, which mostly leveraged enantiopure starting materials^18,20,23^. We also acknowledge that our chemical proteomic datasets were generated with one cell line (Ramos cells) in a basal state and may, therefore, overlook liganding events that occur with dynamic or regulated proteoforms. We should finally note that the original BcpAc stereoprobes (FWG-1A/B, FWG-2A/B, FWG-3A/B and FWG-4A/B) may best serve as tools for generating initial ligandability maps of biological systems. With such maps in hand, medicinal chemistry can then be used to create more potent and selective compounds for functional studies of individual proteins of interest, as we demonstrated through the development of FWG-33B for studying the TRMT112:METTL5 complex. Even with a more optimized chemical probe such as FWG-33B, we recommend including appropriate controls, such as the inactive enantiomer FWG-33A and compound-resistant (for example, METTL5-disrupted) cell models, for full interpretation of on-target pharmacological activity.

Future studies of other cell types and cell states may uncover additional context-dependent proteoform liganding events and, through doing so, further expand the toolbox of chemistries that can perturb biological systems with high precision.

## Methods

### Chemical synthesis

Details on chemical synthesis can be found in Supplementary Information.

### Cell lines and cell culture

Ramos (American Type Culture Collection (ATCC), CRL-1596) cells were grown in RPMI medium supplemented with 10% FBS, 2 mM l-glutamine, penicillin (100 U per ml) and streptomycin (100 μg ml^−1^). 22Rv1 (ATCC, CRL-2505) cells were grown in RPMI medium supplemented with 10% FBS, 2 mM l-alanyl-l-glutamine (GlutaMAX), penicillin (100 U per ml) and streptomycin (100 μg ml^−1^). HEK293T (ATCC, CRL-3216), Lenti-X (Takara, 632180) and HCT116 (ATCC, CCL-247) were grown in DMEM supplemented with 10% FBS, 2 mM l-glutamine, penicillin (100 U per ml) and streptomycin (100 μg ml). Cells were cultured in a humidified, 37 °C tissue culture incubator with 5% CO_2_. All cell lines were routinely inspected for *Mycoplasma* contamination.

### Cloning and mutagenesis

The expression plasmid pRK5_RNF14–FLAG was previously prepared by our lab^75^. Mutagenesis was performed using the Q5 site-directed mutagenesis kit (New England Biolabs (NEB), E0554S) with the following primers.

RNF14-C68A_FWD: ATACACCATTGCCTTTCTGCCTC

RNF14-C68A_REV: TCAAAGCCACTATTCTGG

RNF14-C212A_FWD: GCAGATAAAAGCCTTTAATAGTAAATTGTTCC

RNF14-C212A_FWD: TGAGCTTGATCAAAGTCC

Open reading frames (ORFs) of HA–HEMK2 and HA–BUD23 were synthesized by Integrated DNA Technologies (IDT). The ORF of METTL5 was synthesized by IDT and PCR-amplified (two consecutive PCRs) with primers containing an N-terminal HA tag, using the primers described below.

HA–METTL5_FWD_1: TACCCATACGATGTTCCAGATTACGCTGGTACCATGAAGAAAGTAAGGCTTAA

HA–METTL5_REV_1: CCTAATTCGGTTTTCCTTT

HA–METTL5_FWD_2: GGGGACAAGTTTGTACAAAAAAGCAGGCTGCCACCATGTACCCATACGATGTTCCAG

HA–METTL5_REV_2: ACCACTTTGTACAAGAAAGCTGGGTTCAAAAGGAAAACCGAATTAGGTCC

HA–HEMK2, HA–METTL5 and HA–BUD23 were cloned into a pRK5 expression vector using Gateway technology (Thermo, 11791019 and 11789013) (ORFs in Supplementary Table 3).

### Generation of HCT116 cells stably expressing WT-TRMT112–FLAG, TRMT112-C100A–FLAG or TRMT112-C100S–FLAG

#### Cloning and mutagenesis

Codon-optimized WT-TRMT112–FLAG and TRMT112-C100A–FLAG were synthesized by IDT and cloned into a pLV416 expression vector using Gateway technology (Thermo, 11791019 and 11789013) (ORFs in Supplementary Table 3). The plasmid pLV416_TRMT112-C100S–FLAG was cloned by mutagenesis using a Q5 site-directed mutagenesis kit using the primers described below.

TRMT112-C100S_FWD: AACACTGCAGAGCCCTGAGAGCG

TRMT112-C100S_REV: CCCTCGATCACCTCAAC

#### Virus production

HEK293T cells (6.0 × 10^5^ in 2 ml of DMEM per well) were seeded into six-well dishes and allowed to grow for 1 day. pLV416_WT-TRMT112–FLAG or mutants (1 μg), pCMV-dR8.91 (1 μg) and pCMV-VSV-G (0.1 μg) were mixed in 100 μl of Opti-MEM. Then, 10 μl of 1 mg ml^−1^ polyethylenimine (PEI; Polysciences) was added and incubated for 15 min. The mixture was gently added to the cells. After 8 h, the medium was removed and exchanged with DMEM supplemented with 30% FBS, l-glutamine, penicillin and streptomycin. The virus was collected after 2 and 3 days, combined and filtered with a 0.45-μm syringe (Millipore).

#### Transduction

HCT116 cells (4.0 × 10^5^ cells in 1 ml of DMEM per well) were mixed in a 12-well dish with 200 μl of virus supernatant and 8 μg ml^−1^ polybrene (Millipore). Spinfection was performed (900*g*, 1 h at 30 °C). On day 1, the virus-containing medium was removed and fresh DMEM was added. On day 2, 600 μg ml^−1^ G418 was added to start the selection (2 weeks).

### Generation of HCT116 cells stably expressing WT-TRMT112–FLAG and HA–METTL5

The ORF of HA–METTL5 was cloned into the lentiviral expression vector pLEX307 using Gateway technology (Thermo, 11791019 and 11789013). Virus production and transduction of HCT116_FLAG__–TRMT112_ cells was performed as described above. On day 2 after the transduction, 2 μg ml^−1^ puromycin was added to start the selection (3 days).

### Generation of HCT116 cells with *METTL5* KO

Stable KO cell lines were generated by transduction of HCT116 cells with lentiCRISPR v2 vector (sgRNAs cloned into the vector; Addgene, 52961) using BsmBI (NEB, R0739S) restriction and annealed oligo ligation cloning with the primers below. Virus production transduction of HCT116 cells was performed as described above except with Lenti-X rather than HEK293T cells. On day 2 after the transduction, 2 μg ml^−1^ puromycin was added to start the selection (3 days) and the cells were subsequently expanded for 2 weeks. Successful KO was validated by immunoblotting and genomic DNA sequencing.

sgMETTL5_FWD: CACCGCCAGGCCGCACATTGCAGGT

sgMETTL5_REV: AAACACCTGCAATGTGCGGCCTGGC

### Western blotting

Cells were lysed in cold DPBS by pulse sonication (3 × 8 pulses, 10% power output). Cell lysates were normalized to 1 mg ml^−1^ (DC protein assay), followed by the addition of 4× SDS gel loading buffer (36 μl). Proteins were resolved using SDS–PAGE gel (160 V, 1 h, 4–20% Tris–glycine; Invitrogen, XP04205BOX). Proteins were then blotted onto a PVDF membrane (60 V, 2 h), blocked with 5% milk in Tris-buffered saline with Tween-20 (TBST) for 30 min at room temperature and incubated with primary antibodies (dilutions in Supplementary Table 4) at 4 °C overnight. Membranes were washed three times with TBST before treatment with secondary antibodies for 2 h at room temperature or directly imaged if a primary horseradish peroxidase conjugate was used. Membranes were developed with SuperSignal West Pico PLUS chemiluminescent substrate and visualized by chemiluminescence scan on a ChemiDoc MP imaging system (Bio-Rad).

### Gel-ABPP for proteome-wide reactivity

Ramos cells (5 ml of 3 × 10^6^ cells per ml) were treated with alkyne probes for 1 h. Cells were collected by centrifugation at 500*g* for 5 min at 4 °C and washed twice with cold DPBS. Cell pellets were resuspended in 250 μl of cold DPBS and lysed by pulse sonication (3 × 8 pulses, 10% power output) and normalized to 100 µl of 1 mg ml^−1^ whole-cell lysates (Pierce BCA protein assay). Samples were treated with 11 μl of click master mix (6 µl of 1.7 mM TBTA in 4:1 *t*-butanol and DMSO, 2 µl of 50 mM CuSO_4_ in H_2_O, 2 µl of freshly prepared 50 mM TCEP in H_2_O and 0.8 µl of 1.25 mM TAMRA-PEG3-azide) for 1 h, followed by the addition of 4× SDS gel loading buffer (36 μl). Proteins were resolved by SDS–PAGE (275 V, 4 h, 10% Tris–glycine, made in house) and visualized by in-gel fluorescence on a ChemiDoc MP imaging system (Bio-Rad). The images were processed using Image Lab software (version 6.1.0). Gels were stained with Coomassie InstantBlue protein stain and visualized by Coomassie gel scan on a ChemiDoc MP imaging system (Bio-Rad).

### Gel-ABPP with recombinant RNF14

HEK293T cells (3.5 × 10^5^ in 2 ml of DMEM per well) were seeded into six-well dishes the day before transfection with pRK5_RNF14–FLAG (1 μg) (PEI:DNA = 3:1) for 2 days. Untransfected (UT) cells were treated only with PEI. After 2 days, the medium was exchanged (1 ml of DMEM per well). Cells were treated with DMSO or competitor for 3 h, followed by alkyne for 1 h. Cells were scraped in cold DPBS and collected by centrifugation at 500*g* for 5 min at 4 °C and washed with cold DPBS. The cell pellets were resuspended in 200 μl of cold DPBS and lysed by pulse sonication (3 × 8 pulses, 10% power output). After centrifugation at 21,000*g* for 5 min at 4 °C, the supernatants were normalized to 100 µl of 1 mg ml^−1^ (DC protein assay) and treated with 11 μl of click master mix, as described above. Proteins were resolved by SDS–PAGE gel (160 V, 1 h, 4–20% Tris–glycine; Invitrogen, XP04205BOX) and visualized as described above, followed by western blotting.

### Gel-ABPP with stable HCT116 cell lines

Stable HCT116 cells (7.5 × 10^5^ in 2 ml of DMEM per well) were seeded into six-well dishes the day before treatment. The medium was exchanged (1 ml of DMEM per well) and cells were treated with DMSO or competitor for 3 h, followed by treatment with alkyne for 1 h. Cells were scraped in cold DPBS and collected by centrifugation at 500*g* for 5 min at 4 °C and washed with cold DPBS. The cell pellets were resuspended in 200 μl of cold DPBS and lysed by pulse sonication (3 × 8 pulses, 10% power output). After centrifugation at 21,000*g* for 5 min, the supernatants were normalized to 100 µl of 1 mg ml^−1^ (DC protein assay) and were treated with 11 μl of click master mix, as described above. Proteins were resolved by SDS–PAGE gel (160 V, 1.25 h, 4–20% Tris–glycine) and visualized as described above, followed by western blotting.

#### Modification for transient coexpression with MTs

HCT116_TRMT112__–FLAG_ cells (6.25 × 10^5^ in 2 ml of DMEM per well) were seeded into six-well dishes the day before transfection with pRK5_HA–MT (2.5 μg) (10 μl of Lipofectamine LTX (Invitrogen, 15338100) in 500 μl Opti-MEM (complexed for 30 min) for 1 day. UT cells were treated only with Lipofectamine LTX. Samples were further processed as described as above.

#### Quantification

Band intensities were quantified using Image Lab (version 6.1.0) software (Bio-Rad). IC_50_ curves were generated using GraphPad Prism (version 10.4.1), applying a four-parameter variable slope nonlinear regression with the top and bottom constraints set to 100% and 0%, respectively.

### IP gel-ABPP for TRMT112

HCT116 (parental control) or HCT116_WT-TRMT112__–FLAG_ cells (5.0 × 10^6^ cells in 10 ml of DMEM per dish) were seeded into 10-cm plates the day before. On the day of the treatment, the medium was exchanged (10 ml of DMEM per dish). Cells were treated with DMSO or competitor for 3 h, followed by treatment with alkyne for 1 h and harvesting. Cells were scraped in cold DPBS, collected by centrifugation at 500*g* for 5 min at 4 °C and washed with cold DPBS. The cell pellets were resuspended in 500 μl of cold DPBS containing 1% Nonidet P-40 (IGEPAL CA-630, Sigma-Aldrich, I8896) and cOmplete protease inhibitor cocktail (Roche) and lysed by pulse sonication (eight pulses, 10% power output). The lysate was centrifuged at 21,000*g* for 5 min.

#### For the input

As input, 50 µl of 1 mg ml^−1^ of normalized supernatants (DC protein assay) were treated with 5.5 μl of click master mix and processed as described above.

#### For the IP

First, 500 μl of 2 mg ml^−1^ of supernatants were incubated with washed Pierce anti-DYKDDDDK magnetic agarose (Thermo, A36797; 40 μl of 25% slurry and sample) for 2 h at 4 °C with rotation. After incubation, the beads were isolated with a magnetic stand and washed three times with 0.2% Nonidet P-40 in DPBS (1 ml), followed by once with DPBS (1 ml). The beads were resuspended in 50 μl of DPBS and treated with 5.5 μl of click master mix and further processed as described above, followed by western blotting.

### GSH reactivity assay

As described previously^23^, GSH was diluted to a final concentration of 50 µM in 0.1 M Tris-HCl pH 8.8 and 30% acetonitrile. In triplicate, 100 µl of the GSH solution was added to a clear 384-well plate (Greiner, 781101). Stereoprobes (5 µl of 10 mM) were then added to the GSH solution to achieve a final probe concentration of 500 µM and the reaction was incubated for 6 h at room temperature. Ellman’s reagent (5 µl of 100 mM) was then added to the plate and the absorbance was read at 440 nm. The concentration of GSH remaining was derived from a standard curve and the observed rate (*k*_obs_/[*I*]) was calculated assuming pseudo-first-order reaction kinetics from the following equations:

1 $${\rm{d}}[\mathrm{GSH}]/{\rm{d}}t=-k_{\rm{obs}}\times [\mathrm{GSH}]$$

2 $$[\mathrm{GSH}]_t=[\mathrm{GSH}]_{\rm{t0}}\times {e}^{{-k}_{\rm{obs}}t}$$

### Protein-directed ABPP

#### In situ treatment and sample processing

As described previously^23^, Ramos cells (10 ml of 3 × 10^6^ cells per ml) were treated with DMSO or competitor for 3 h, followed by treatment with a stereomatched alkyne for 1 h. The cells were harvested on ice, washed twice with cold DPBS and stored at −80 °C. The cell pellets were resuspended in 500 μl cold DPBS and lysed by pulse sonication (3 × 8 pulses, 10% power output). Then, 500 µl of 1 mg ml^−1^ of normalized whole-cell lysates (Pierce BCA protein assay) were treated with 55 μl of click MS master mix (30 µl of 1.7 mM TBTA in 4:1 *t*-butanol and DMSO, 10 µl of 50 mM CuSO_4_ in H_2_O, 10 µl of freshly prepared 50 mM TCEP in H_2_O and 10 µl of 10 mM biotin-PEG4-azide). Proteins were precipitated with cold methanol (600 μl), chloroform (200 μl) and water (100 μl), vortexed and then centrifuged at 16,000*g* for 10 min. The top and bottom layers were aspirated and the protein disk was sonicated in 500 μl of methanol and pelleted at 16,000*g* for 10 min. After the methanol was completely aspirated, protein pellets were immediately processed or stored at −80 °C. Pellets were resuspended in 500 μl of freshly prepared 8 M urea in DPBS, followed by the addition of 10 μl of 10 wt.% SDS. Samples were then pulse-sonicated until clear. The samples were reduced with 25 μl of 200 mM dithiothreitol (DTT) at 65 °C for 15 min, followed by alkylation with 25 μl of 400 mM IA at 37 °C for 30 min. Then, 65 μl of 20 wt.% SDS was added and the samples were transferred to a 15-ml tube in a total volume of 6 ml with DPBS (0.2% final SDS). Washed streptavidin beads (Thermo, 20353; 100 μl of 50% slurry and sample) were then added and proteins were enriched for 1.5 h at room temperature with rotation. After incubation, the beads were pelleted (2 min at 2,000*g*) and washed with 0.2 wt.% SDS in DPBS (twice, 10 ml each), DPBS (once, 5 ml), high-performance LC (HPLC)-grade water (twice, 1 ml each) and 200 mM EPPS (1 ml, pH 8.0). Enriched proteins were digested on beads overnight with 200 μl of trypsin mix (2 M urea, 1 mM CaCl_2_, 10 μg ml^−1^ trypsin (Promega, V5111) and 200 mM EPPS, pH 8.0). The beads were pelleted at 2,000*g*; the supernatant was collected and then diluted with 100 μl of acetonitrile (30% final). Samples were then labeled with 6 μl of 20 mg ml^−1^ (in dry acetonitrile) TMTpro 16plex tag (Thermo, A44520) or TMT 10plex (Thermo, 90406) for 1.5 h at room temperature, vortexing every 30 min. TMT labeling was quenched by the addition of hydroxylamine (6 μl of 5% solution in H_2_O) and incubated for 15 min at room temperature. Samples were then acidified with 20 μl of formic acid, combined and dried using a SpeedVac at 46 °C. Samples were desalted with a Sep-Pak column Vac 1 cm^3^ (50 mg) (Waters, WAT054955) and then fractionated at high pH into 10 fractions (for 16plex) or 5 fractions (10plex) using Pierce peptide desalting spin columns (Thermo, 89852) and an acetonitrile–NH_4_HCO_3_ (10 mM) gradient (Supplementary Tables 5 and 6) and analyzed by MS as described below.

#### Modifications for adherent cells

##### Protein-directed ABPP for RNF14

HEK293T cells (3.5 × 10^6^ in 10 ml of DMEM) were seeded into four 10-cm plates the day before transfection with pRK5_RNF14–FLAG (5 μg per dish) (PEI:DNA = 3:1). After 1 day, the cells were pooled and distributed over 18 10-cm plates with 10 ml of DMEM each. The next day, the medium was exchanged (7 ml of DMEM per dish) and cells were treated with DMSO or competitor for 3 h, followed by treatment with alkyne for 1 h. Cells were scraped in medium on ice, washed twice with cold DPBS and stored at −80 °C. The samples were further processed as described above.

##### Comparative protein-directed ABPP (parental versus KO cells)

HCT116 or HCT116_*METTL5*-KO_ cells (4.5 × 10^6^ in 10 ml of DMEM per dish) were seeded into 10-cm plates the day before. On the day of the treatment, the medium was exchanged (7 ml of DMEM per dish). Cells were treated with alkyne for 1 h. Cells were scraped in cold DPBS, washed twice with cold DPBS and stored at −80 °C. The samples were further processed as described as above.

##### Comparative protein-directed ABPP (suspension version adherent cells)

First, 22Rv1 cells (15 × 10^6^ in 25 ml of RMPI medium per dish) were seeded into 15-cm plates the day before. On the day of the treatment, the medium was exchanged (13 ml of DMEM per dish). The 22Rv1 cells and Ramos cells (13 ml of 3 × 10^6^ cells per ml) were treated with DMSO or competitor for 3 h, followed by treatment with alkyne for 1 h. The 22Rv1 cells were scraped in DPBS and Ramos cells were harvested in medium on ice. The cells were washed twice with cold DPBS and stored at −80 °C. The samples were further processed as described above.

#### TMT LC–MS analysis

Fractions were resuspended in buffer A (5% acetonitrile and 0.1% formic acid in water) and analyzed by TMT LC–MS using an Orbitrap Fusion Tribrid MS instrument (Thermo Scientific) coupled to an UltiMate 3000 Series rapid separation LC system and autosampler (Thermo Scientific Dionex). The peptides were eluted onto a capillary column (inner diameter: 75 μm, fused silica, packed with C18; Waters, Acquity BEH C18, 1.7 μm, 25 cm) or an EASY-Spray HPLC column (Thermo, ES902 and ES903) using an Acclaim PepMap 100 (Thermo, 164535) loading column and separated at a flow rate of 0.25 μl min^−1^. Peptides were separated across a 10-min gradient of 5%, 150-min gradient of 5–20%, 20-min gradient of 20–45% and then 5-min gradient of 45–95% acetonitrile (0.1% formic acid) in H_2_O (0.1% formic acid) followed by column equilibration. Data were acquired using an MS3-based TMT method on Orbitrap Fusion or Eclipse Tribrid MS instruments.

For the Orbitrap Fusion, the scan sequence began with an MS1 master scan (Orbitrap analysis, resolution: 120,000, 400–1,700 *m*/*z*, radiofrequency (RF) lens: 60%, maximum injection time: 50 ms) with dynamic exclusion enabled (repeat count: 1, duration: 15 s). The top precursors were then selected for MS2 and MS3 analysis. MS2 analysis consisted of quadrupole isolation (isolation window: 0.7) of precursor ion followed by collision-induced dissociation in the ion trap (collision energy: 35%, maximum injection time: 120 ms). Following the acquisition of each MS2 spectrum, synchronous precursor selection enabled the selection of up to ten MS2 fragment ions for MS3 analysis. MS3 precursors were fragmented by higher-energy collisional dissociation (HCD) and analyzed using the Orbitrap (collision energy: 55, maximum injection time: 120 ms, resolution: 50,000). For MS3 analysis, we used charge-state-dependent isolation windows. For charge state *z* = 2, the MS isolation window was set at 1.2; for *z* = 3–6, the MS isolation window was set at 0.7.

For the Eclipse Tribrid, the scan sequence began with an MS1 master scan (Orbitrap analysis, resolution: 120,000, 400–1,700 *m*/*z*, RF lens: 30%, maximum injection time: 50 ms) with dynamic exclusion enabled (repeat count: 1, duration: 30 s). The top precursors were then selected for MS2 and MS3 analysis. MS2 analysis consisted of quadrupole isolation (isolation window: 0.7) of precursor ion followed by HCD in the ion trap (collision energy: 36%, maximum injection time: 120 ms). Following the acquisition of each MS2 spectrum, synchronous precursor selection enabled the selection of up to ten MS2 fragment ions for MS3 analysis. MS3 precursors were fragmented by HCD and analyzed using the Orbitrap (collision energy: 55%, maximum injection time: 120 ms, resolution: 30,000). For MS3 analysis, we used charge-state-dependent isolation windows. For charge state *z* = 2, the MS isolation window was set at 1.2; for *z* = 3, the MS isolation window was set at 0.7; for *z* = 4–6, the MS isolation window was set at 0.4.

#### Data processing

Raw files were uploaded to the Integrated Proteomics Pipeline (version 6.7.1; http://ip2.scripps.edu/ip2/mainMenu.html). MS2 and MS3 files were extracted from the raw files using RAW Converter (version 1.1.0.22; http://fields.scripps.edu/rawconv/) and searched using the ProLuCID algorithm using a reverse-concatenated, nonredundant variant of the Human UniProt database (release 2016-07). Cysteine residues were searched with a static modification for carboxyamidomethylation (+57.02146 Da). N termini and lysine residues were also searched with a static modification corresponding to the TMT tag (+229.1629 Da for 10-plex and +304.2071 Da for 16-plex). Peptides were required to be at least 6 a.a. long. ProLuCID data were filtered through DTASelect (version 2.0) to achieve a spectrum false-positive rate below 1%. We included a keratin filter, excluded nonunique peptides and required at least one tryptic cleavage site and two peptides per protein. The MS3-based peptide quantification was performed with reporter ion mass tolerance set to 20 ppm with the Integrated Proteomics Pipeline.

#### Data analysis

Enrichment ratios (probe versus probe) were calculated for each peptide–spectrum match (PSM) by dividing the TMT reporter ion intensity by the total intensity across all channels. PSMs were grouped by protein ID, excluding peptides with summed reporter ion intensities <10,000, signal-to-noise ratio <1.0 or isolation purity <0.5. Proteins supported by fewer than two peptides were also excluded. For two experiments, one TMT channel showed substantially reduced reporter ion intensity and was excluded from analysis. To maintain 16-channel balance during normalization, the corresponding replicate channel was temporarily duplicated and protein signals were normalized by total intensity across all channels (sum = 100), including the duplicated channel. The temporarily duplicated channel was finally removed before any downstream statistical analysis.

Replicate channels were grouped across each experiment and mean values were calculated for each protein. To assess measurement variability, the coefficient of variation (CV), defined as the ratio of the s.d. to the mean, was calculated across replicate channels. Proteins with a CV ≥ 0.2 in the most enriched (DMSO + alkyne) channel were excluded from further analysis.

#### Criteria for liganding in protein-directed ABPP

Proteins were initially defined as liganded if they exhibited more than twofold enantioselective enrichment and >50% competition. All proteins passing the initial filters for liganding were manually reviewed to remove proteins showing additional evidence of high variability.

### Cysteine-directed ABPP

#### In situ treatment and sample processing

As described previously^23^, Ramos cells (10 ml of 3 × 10^6^ cells per ml) were seeded 30 min before the experiment. Cells were treated with DMSO or competitor for 3 h. The cells were harvested on ice, washed twice with cold DPBS and stored at −80 °C. The cell pellets were resuspended in 500 μl of cold DPBS and lysed by pulse sonication (3 × 8 pulses, 10% power output). The total protein content of whole-cell lysates was measured using a Pierce BCA protein assay kit and the samples were normalized to 2 mg ml^−1^ and 500 μl. Samples were treated with 5 μl of 10 mM IA-DTB (in DMSO) for 1 h at room temperature, with vortexing every 20 min. Proteins were precipitated by the addition of cold methanol (600 μl), chloroform (200 μl) and HPLC-grade water (100 μl), followed by vortexing and centrifugation at 16,000*g* for 10 min. Without disrupting the protein disk, both the top and the bottom layers were aspirated and the protein disk was sonicated again in 500 μl of methanol and centrifuged at 16,000*g* for 10 min. After the methanol was completely aspirated, protein pellets were immediately processed or frozen at −80 °C. Pellets were resuspended in 90 μl of denaturing and reducing buffer (9 M urea, 10 mM DTT and 50 mM TEAB, pH 8.5). The samples were reduced by heating at 65 °C for 20 min, followed by alkylation with 10 μl of 500 mM IA at 37 °C for 30 min. The samples were then centrifuged at 16,000*g* for 2 min to pellet any insoluble precipitate and probe-sonicated once more to ensure complete resuspension and then diluted with 300 μl of 50 mM TEAB pH 8.5 to reach a final urea concentration of 2 M. Trypsin (4 μl of 0.25 μg μl^−1^ in trypsin resuspension buffer with 25 mM CaCl_2_) was added to each sample and digested at 37 °C overnight. Digested samples were then diluted with 300 μl of enrichment buffer (50 mM TEAB pH 8.5, 150 mM NaCl and 0.2% Nonidet P-40 (IGEPAL CA-630, Sigma-Aldrich, I8896)) containing streptavidin–agarose beads (50 μl of 50% slurry and sample) and were rotated at room temperature for 2 h. The samples were centrifuged (2,000*g*, 2 min) and the entire content was transferred to BioSpin columns and washed (three times with 1 ml of wash buffer, three times with 1 ml of DPBS and three times with 1 ml of water). Enriched peptides were eluted from the beads with 300 μl of 50% acetonitrile with 0.1% formic acid and dried using a SpeedVac at 46 °C. Enriched peptides were resuspended in 100 μl of EPPS buffer (200 mM, pH 8.0) with 30% acetonitrile, vortexed and sonicated in a water bath. The samples were TMT-labeled by the addition of 3 μl of 20 mg ml^−1^ (in dry acetonitrile) of corresponding TMT 10plex tag for 1.5 h at room temperature, with vortexing every 30 min. TMT labeling was quenched by the addition of hydroxylamine (3 μl of 5% solution in H_2_O) and incubated for 15 min at room temperature. Samples were then acidified with 5 μl of formic acid, combined and dried using a SpeedVac. Samples were desalted with a Sep-Pak column, fractionated at high pH by HPLC (described in the following) into a 96-well plate and recombined into 12 fractions (total).

As previously described^23^, the cysteine-directed ABPP samples were resuspended in 500 μl of buffer A and fractionated with an Agilent HPLC system into a 96-deep-well plate containing 20 μl of 20% formic acid to acidify the eluting peptides. The peptides were eluted onto a capillary column (Zorbax 300Extend-C18, 3.5 μm) and separated at a flow rate of 0.5 ml min^−1^ using an acetonitrile–NH_4_HCO_3_ (10 mM) gradient (Supplementary Table 7). Peptides were collected in 300-μl fractions from minutes 5–75. The plates were evaporated to dryness using a SpeedVac; peptides were resuspended in 80% acetonitrile with 0.1% formic acid and combined for a total of 12 fractions (Supplementary Table 8). Samples were dried with a SpeedVac and analyzed by MS as described above.

#### Data processing

Raw files were processed as described above with one modification. A dynamic modification for IA-DTB labeling (+398.25292 Da) was included with a maximum number of two differential modifications per peptide. We included a keratin filter, accepted nonunique peptides and required at least one tryptic cleavage site.

#### Data analysis

Cysteine engagement ratios (DMSO versus compound) were calculated for each PSM by dividing each TMT reporter ion intensity by the average intensity for the DMSO channels. PSMs were then grouped on the basis of protein ID and residue number, excluding peptides with summed reporter ion intensities for the two DMSO channels of <10,000 and a CV for DMSO channels of >0.5 in each experiment. Replicate channels were grouped across each experiment and average values were computed for each cysteine site and peptides with mean absolute deviation or mean >0.2 were excluded.

#### Criteria for liganding in cysteine-directed ABPP

Sites were initially defined as liganded if they exhibited >50% competition, more than twofold enantioselectivity and at least one of the following additional criteria to distinguish liganding from changes driven by protein abundance.

If the protein passed the ‘liganded’ criteria in protein-directed ABPP, then detection of liganding at a single site was considered sufficient.

If the protein did not pass the ‘liganded’ criteria in protein-directed ABPP, we required evidence of at least one additional peptide that did not show liganding by cysteine-directed ABPP to classify the site as liganded.

All proteins passing the initial filters for liganding were manually reviewed to remove proteins showing additional evidence of high variability. Liganded peptides were required to be unique.

#### Modifications for cysteine-directed ABPP for RNF14 with Glu-C digestion

HEK293T cells (3.5 × 10^6^ in 10 ml of DMEM) were seeded into five 10-cm plates the day before transfection with pRK5_RNF14–FLAG (5 μg per dish) (PEI:DNA = 3:1). After 1 day, the cells were pooled and distributed over 11 15-cm plates with 25 ml of medium each. The following day, the medium was exchanged (13 ml of DMEM per dish) and cells were treated with DMSO or competitor for 3 h. Cells were scraped in medium on ice, washed twice with cold DPBS and stored at −80 °C. The cell pellets were resuspended in 500 μl of cold DPBS and lysed by pulse sonication (3 × 8 pulses, 10% power output). The lysate was cleared by ultracentrifugation at 100,000*g* for 30 min. Normalized clarified lysates (2 mg ml^−1^ and 500 μl) were further processed as described above with some modifications. After methanol precipitation, the samples were resuspended in 90 μl of denaturing and reducing buffer (8 M urea, 10 mM DTT and 50 mM NH_4_HCO_3_). After reduction and alkylation, 800 μl of 50 mM NH_4_HCO_3_ was added (final urea concentration: 0.8 M). Glu-C (4 μg) (Promega, V1651) was added to each sample and digested at 37 °C overnight. Digested samples were then diluted with 300 μl of enrichment buffer (50 mM TEAB pH 8.5, 150 mM NaCl and 0.3% Nonidet P-40) containing streptavidin–agarose beads (50 μl of 50% slurry and sample) and further processed as described above. In the data-processing step, we searched with C-terminal aspartic or glutamic acid cleavage instead of C-terminal lysine or arginine cleavage.

### IP–MS experiments for TRMT112

#### In situ treatment and sample processing

HCT116 (parental control) or stable HCT116 cells (5.0 × 10^6^ in 10 ml of DMEM per dish) were seeded into 10-cm plates the day before. On the day of the treatment, the medium was exchanged (10 ml of DMEM per dish). Cells were treated with DMSO or competitor for 4 h. Cells were scraped in cold DPBS, washed twice with cold DPBS and stored at −80 °C. The cell pellets were resuspended in 500 μl of lysis buffer (50 mM EPPS pH 7.5 and 150 mM NaCl) containing 1% Nonidet P-40 (IGEPAL CA-630, Sigma-Aldrich, I8896) and cOmplete protease inhibitor cocktail (Roche) and lysed by rotation at 4 °C for 1 h. After centrifugation at 21,000*g* for 5 min, 500 µl of 2 mg ml^−1^ normalized supernatant (BCA protein assay kit) was incubated with washed Pierce anti-DYKDDDDK magnetic agarose (Thermo, A36797; 40 μl of 25% slurry and sample) or Pierce anti-HA magnetic beads (Thermo, A36797; 40 μl of slurry and sample) for 2 h at 4 °C with rotation. After incubation, the beads were isolated with a magnetic stand and washed three times with 1 ml of wash buffer (25 mM EPPS pH 7.5 and 150 mM NaCl) containing 0.2% Nonidet P-40, followed by once with 1 ml of 50 mM EPPS pH 8.0. Proteins were eluted from beads in 40 μl of 8 M urea in DPBS at 65 °C for 10 min. The samples were reduced with 2 μl of 200 mM DTT at 65 °C for 15 min, followed by alkylation with 2 μl of 400 mM IA at 37 °C for 30 min. The total volume was brought to 160 μl with 50 mM EPPS pH 8 (final urea concentration: 2 M). Enriched proteins were digested overnight 37 °C with trypsin (1 μl of 100 mM CaCl_2_ and 1 μg of trypsin per sample). Then, 70 μl of acetonitrile (30% final) was added, followed by 6 μl of 20 mg ml^−1^ (in dry acetonitrile) of the corresponding TMTpro 16plex tag for 1.5 h at room temperature, with vortexing every 30 min. TMT labeling was quenched by the addition of hydroxylamine (6 μl of 5% solution in H_2_O) and incubated for 15 min at room temperature. Samples were then acidified with 5 μl of formic acid, combined and dried using a SpeedVac at 46 °C. Samples were desalted with a Sep-Pak column and then fractionated at high pH into three fractions using peptide desalting spin columns and acetonitrile–NH_4_HCO_3_ (10 mM) gradient into three fractions (Supplementary Table 9) and analyzed by MS.

#### Data processing

Raw files were processed as described for the protein-directed samples above.

### SEC–ABPP

HCT116_WT-TRMT112__–FLAG_ cells (10 × 10^6^ in 25 ml of DMEM per dish) were seeded into 15-cm plates the day before. On the day of the treatment, the medium was exchanged (13 ml of DMEM per dish). Cells were treated with alkyne for 1 h (comparative SEC–ABPP) or pretreated with DMSO or competitor for 3 h (competitive SEC–ABPP), followed by alkyne for 1 h. Cells were scraped in cold DPBS and washed twice with cold DPBS. The cell pellets were resuspended in cold DPBS (600 μl) containing cOmplete protease inhibitor cocktail (Roche) and PhosSTOP phosphatase inhibitor cocktail (Roche) and lysed by pulse sonication (1 × 8 pulses, 10% power output). The lysate was cleared by ultracentrifugation 100,000*g* for 30 min. Normalized clarified lysates (2 mg ml^−1^, DC protein assay) were filtered using a centrifugal filter (Milipore, UFC30GV; 20 μM PVDF) and 500 μl of clarified lysate was injected into a Superdex 200 Increase 10/300 GL column attached to an ÄKTA pure fast protein LC (FPLC) system (Cytiva). Proteins were fractionated using an isocratic gradient (DPBS) running at 0.5 ml min^−1^ into five 2-ml fractions, beginning at 8 ml and ending at 18 ml. Eluate was collected into 15-ml tubes containing 0.5 ml of PBS with 1% Nonidet P-40 (IGEPAL CA-630, Sigma-Aldrich, I8896) and cOmplete protease inhibitor cocktail (Roche). SEC fractions were incubated with washed anti-FLAG M2 affinity gel (Sigma-Aldrich, A2220; 60 μl of 50% slurry and sample) overnight at 4 °C with rotation. After incubation, the beads were pelleted (2 min at 2,000*g*) and washed three times with 1 ml of 0.2% Nonidet P-40 in DPBS, followed by once with 1 ml of DPBS. The beads were resuspended in 50 μl of DPBS and treated with 5.5 μl of click master mix for 1 h, followed by the addition of 4× SDS gel loading buffer (18 μl). Proteins were resolved by SDS–PAGE gel (160 V, 1.25 h, 4–20% Tris–glycine) and visualized as described above, followed by western blotting.

### Abundance-based SEC analysis

HCT116_TRMT112__–FLAG_ cell pellets were seeded the day before the experiment, scraped in cold DPBS, washed twice with cold DPBS and processed as described for SEC–ABPP. The eluate from the FPLC was collected into tubes containing each 12 ml of acetone at 4 °C. Proteins were precipitated overnight at −20 °C and then centrifuged at 4,500*g* for 20 min; the acetone–DPBS mixture was decanted off the pellets. The samples were dried at room temperature and resuspended in 1× SDS gel loading buffer (100 μl), followed by sonication in a water bath and heating to 98 °C for 5 min. Proteins were resolved by SDS–PAGE gel (160 V, 1.25 h, 4–20% Tris–glycine) and visualized as described above, followed by western blotting.

### Protein purification

#### Cloning

The full-length human *TRMT112* and *METTL5* genes were codon-optimized for *E*. *coli* cell expression were synthesized by IDT. The ORF of *TRMT112* was cloned into a pACYCDuet-1 vector using NdeI (NEB, R0111S) and BglII (NEB, R0144S) restriction and Gibson assembly (NEB, E2621S). Mutagenesis was carried out using a Q5 site-directed mutagenesis kit (NEB, E0554S) with the following primers:

TRMT112-C100Α_FWD: AACTTTGCAGGCGCCAGAATCTGGG

TRMT112-C100Α_REV: CCCAGATTCTGGCGCCTGCAAAGTT

The ORF of *METTL5* was synthesized by IDT and was PCR-amplified with primers containing an N-terminal His tag followed by a TEV protease cleavage site (ORFs in Supplementary Table 3). The PCR product was cloned into a pET21a vector using NdeI (NEB, R0111S) and NotI (NEB, R0189S) restriction and Gibson assembly (NEB, E2621S).

His–METTL5_Gibson_FWD: taactttaagaaggagatatacATATGCACCACCACCACCACCACGAAAATTTGTATTTCCAGTCAATGAAGAAGGTACGTCTG

His–METTL5_Gibson_REV: GGTGGTGCTCGAGTGCGGCCGCTCAAAAACTGAAGCGGATCA

#### Protein expression of TRMT112:His–METTL5

With modifications to a previously described procedure^38^, the TRMT112 and His–METTL5 complex was coexpressed in *E*. *coli* BL21 (DE3). *E*. *coli* was grown in 1 L of culture in autoinducible Terrific Broth (Formedium, AIMTB0260) containing carbenicillin (50 μg ml^−1^) and chloramphenicol (34 μg ml^−1^). The culture was initially incubated at 37 °C for 3.5 h and then at 18 °C overnight. The cells were harvested by centrifugation at 4,000*g* for 30 min and flash-frozen in liquid nitrogen. Cell pellets were resuspended in 30 ml of cold lysis buffer (50 mM Tris-HCl pH 7.5, 200 mM NaCl, 1 mM TCEP with cOmplete protease inhibitor cocktail (Roche) and lysozyme). The cells were lysed by pulse sonication on ice and the lysate was cleared by centrifugation at 20,000*g* for 45 min. The supernatant was incubated with prewashed HisPur Ni-NTA resin (Thermo, 88221; 1 ml of 50% slurry) and 10 mM imidazole at 4 °C for 1 h with rotation. The beads were washed in a gravity-flow column with 10 ml of cold wash buffer 1 (50 mM Tris-HCl pH 7.5, 1 M NaCl, 1 mM TCEP and 10 mM imidazole), 10 ml of wash buffer 2 (50 mM Tris-HCl pH 7.5, 200 M NaCl, 1 mM TCEP and 10 mM imidazole) and 10 ml of wash buffer 3 (50 mM Tris-HCl pH 7.5, 200 M NaCl, 1 mM TCEP and 20 mM imidazole). Proteins were eluted from beads with 5 ml of elution buffer (50 mM Tris-HCl pH 7.5, 200 M NaCl, 1 mM TCEP and 400 mM imidazole). Proteins were dialyzed using a Slide-A-Lyzer dialysis cassettes (10-kDa molecular weight cutoff (MWCO); Thermo, 66830) into anion-exchange buffer A (50 mM Tris-HCl pH 7.5, 100 mM NaCl and 0.5 mM TCEP) overnight at 4 °C.

Proteins were injected into a HiTrap Q FF anion-exchange chromatography column (Cytiva, 17515601) attached to an ÄKTA pure FPLC system (Cytiva). Proteins were fractionated using a linear gradient from anion-exchange buffer A to anion-exchange buffer B (50 mM Tris-HCl pH 7.5, 1 M NaCl and 0.5 mM TCEP) over eight column volumes at 5 ml min^−1^. Eluate was collected in 500-μl fractions. TRMT112:METTL5-containing fractions were combined, concentrated with an Amicon Ultra centrifugal filter (3-kDa MWCO; Milipore, UFC900308) and then injected into a Superdex 200 Increase 10/300 GL column attached to an ÄKTA pure FPLC system (Cytiva). Proteins were fractionated using an isocratic gradient (50 mM Tris-HCl pH 7.5, 200 mM NaCl and 1 mM TCEP) running at 0.5 ml min^−1^ and collected in 500-μl fractions. TRMT112:METTL5-containing fractions were combined, quantified by NanoDrop and flash-frozen in liquid nitrogen. Typical yields were 5–10 mg of protein complex per 1 L of culture.

#### Protein expression of tag-free TRMT112:METTL5

Tag-free TRMT112:METTL5 was expressed from the same constructs as described above. After the dialysis step, 0.5 mg of His-tagged TEV protease (gift from Vividion Therapeutics) was added and the protein was incubated at 4 °C for 1 day. The protein was then incubated with prewashed HisPur Ni-NTA resin (Thermo, 88221; 1 ml of 50% slurry) at 4 °C for 1 h with rotation. The supernatant was filtered using a gravity-flow column and further processed as described above. A Coomassie-stained gel of all purification steps is shown in Supplementary Fig. 3.

### Intact protein MS and rate determination

Purified 0.5 μΜ TRMT112:His–METTL5 complex was incubated with 10 μΜ FWG-33B in 50 mM Tris-HCl pH 7.5, 200 mM NaCl, 1 mM DTT and 5% DMSO. Reactions were stopped after 10 and 60 min by the addition of formic acid.

The observed rate (*k*_obs_/[I]) was calculated assuming pseudo-first-order reaction kinetics:

3 $${\rm{d}}[{\mathrm{TRMT}}112{{:}}{\mathrm{METTL}}5]/{\rm{d}}t=-k_{\rm{obs}}\times [{\mathrm{TRMT}}112{{:}}{\mathrm{METTL}}5]$$

4 $$[{\mathrm{TRMT}}112{{:}}{\mathrm{METTL}}5]_{t}=[{\mathrm{TRMT}}112{{:}}{\mathrm{METTL}}5]_{{\rm{t0}}}\times {e}^{{-k}_{\rm{obs}}t}$$

Samples were analyzed on an Agilent LC1290 Infinity II instrument coupled to an Agilent 6545 quadrupole time-of-flight LC–MS instrument (Agilent Technologies). A sample volume of 10 μl was injected. The protein was desalted and separated on an AERIS 3.6-μm-wide-bore XB-C8 LC column (50 × 2.1 mm^2^; Phenomenex) at 60 °C with a flow rate of 0.5 ml min^−1^. Mass spectra were acquired from 700 to 1,700 Da at a resolution of 25,000. A dual Agilent Jet Stream electrospray ionization (ESI) source was used for ionization. The resulting data files were deconvoluted to protein masses using Agilent MassHunter BioConfirm software (version 11.0).

### Protein crystallization and structure determination

Tag-free 20 μΜ TRMT112:METTL5 complex was incubated with 100 μΜ FWG-33B in 50 mM Tris-HCl pH 7.5, 200 mM NaCl, 1 mM TCEP, 2% DMSO and 1 mM SAM (Sigma-Aldrich, A7007) and the reaction was monitored by intact protein MS. Upon completion (<30 min), the protein sample was buffer-exchanged into 50 mM Tris-HCl pH 7.5, 200 mM NaCl and 1 mM TCEP using a PD-10 desalting column (Cytiva, 17085101) and concentrated to 24 mg ml^−1^. Initial crystals were identified in numerous conditions but diffracted poorly. These were used for matrix microseeding into commercial screens. Crystals used in diffraction experiments were grown in drops consisting of 1:1, 1:2 and 2:1 ratios of protein to reservoir solution equilibrated against 21% PEG3350, 0.36 M ammonium sulfate and 0.1 M Bis–Tris pH 5.5 at 4 °C. Crystals were cryoprotected by rapid transfer into reservoir solution supplemented with 30% glycerol before flash-freezing in liquid nitrogen. Diffraction data were collected on Advanced Light Source beamline 5.0.2 and processed with XDS. The structure was determined by molecular replacement in Phaser using TRMT112 and METTL5 from PDB 6H2U as independent search models. The structure was refined using iterative rounds of refinement in REFMAC5 with manual inspection and model building in Coot. Ligand restraints for FWG-33B and the covalent bond with C100 were generated in JLigand. Waters were automatically added in Coot and REFMAC5 and manually inspected. Data collection and refinement statistics can be found in Supplementary Table 2.

### Binding-site mapping of TRMT112 complexes

Binding-site detection was performed on crystallographic structures using SiteMap on Schrödinger Maestro (version 13.2.128; MMshare version 5.8.128, release 2022-2, platform Darwin-x86_64). Structures were generated in Chimera-X (version 1.9).

### Gel-ABPP with purified TRMT112:METTL5 with competitors

First, 50 μl of purified 1 μΜ TRMT112:His–METTL5 complex in DPBS containing 1 mM TCEP and 1 mM SAM was treated with DMSO or competitor for 1 h (2% final DMSO), followed by treatment with alkyne (1 μM) for 1 h. Then, 5.5 μl of click master mix was added, as described above. Proteins were resolved by SDS–PAGE gel (160 V, 1.25 h, 4–20%) and visualized as described above.

### Gel-ABPP with purified TRMT112:METTL5 under kinetic controlled alkyne labelling

First, 350 μl of purified 1 μΜ TRMT112:His–METTL5 complex in DPBS containing 1 mM TCEP was treated with alkyne (0.5 μM, 2% final DMSO and 1 μΜ final protein). At the indicated time points, 50 μl of the reaction mixture was mixed with 5.5 μl of click master mix, as described above. Proteins were resolved by SDS–PAGE gel (160 V, 1.25 h, 4–20%) and visualized as described above.

### Gel-ABPP with purified TRMT112:METTL5 for IC_50_ determination

First, 48 μl of purified 1.04 μΜ TRMT112:His–METTL5 complex in DPBS containing 1 mM TCEP was treated with FWG-33B for 1 h (1% DMSO), followed by treatment with alkyne (0.5 μM, 2% final DMSO, 1 μΜ final protein) for 10 min. Then, 5.5 μl of click master mix was added, as described above. Proteins were resolved by SDS–PAGE gel (160 V, 1.25 h, 4–20%) and visualized as described above.

### Gel-ABPP with nonelectrophilic propanamide

First, 48 μl of purified 1.04 μΜ TRMT112:His–METTL5 complex in DPBS containing 1 mM TCEP was treated with FWG-33B or FWG-69B for 1 h (1% DMSO), followed by treatment with alkyne (0.5 μM, 2% final DMSO and 1 μΜ final protein) for 10 min. Then, 5.5 μl of click master mix was added, as described above. Proteins were resolved by SDS–PAGE gel (160 V, 1.25 h, 4–20%) and visualized as described above.

#### Quantification

Band intensities were quantified using Image Lab (version 6.1.0) software (Bio-Rad). IC_50_ curves were generated using GraphPad Prism (version 10.4.1), applying a four-parameter variable slope nonlinear regression with the top and bottom constraints set to 100% and 0%, respectively.

### In vitro MT assay (LC–MS)

rRNA probes (biotinylated 12-nt and 60-nt substrate; Fig. 5b) were synthesized by IDT.

Purified 20 μΜ WT-TRMT112:His–METTL5 or TRMT112-C100A:His–METTL5 complexes were incubated with 40 μΜ FWG-33B in reaction buffer (50 mM Tris-HCl pH 7.5, 200 mM NaCl, 1 mM DTT, 5% DMSO and 1 mM SAM (Sigma-Aldrich, A7007)) for 30 min at room temperature. Then, 1 μl (20×, final concentration: 1 μM) of this protein mixture was diluted into 19 μl of the substrate assay (final concentrations: 50 mM Tris-HCl pH 7.5, 50 mM NaCl, 5 mM MgCl_2_, 1 mM DTT, 1 U of SUPERase·In RNase inhibitor (Invitrogen, AM2694), 10 μM 12-nt RNA probe and 1 mM SAM) for 1 or 3 h at 37 °C. Each enzymatic assay was performed in triplicate from a single treatment (referred to as technical replicate). The samples were then diluted with 350 μl of cold IP buffer (50 mM Tris-HCl pH 7.5, 150 mM NaCl and 0.1% Nonidet P-40) and incubated with prewashed Dynabeads MyOne Streptavidin T1 (Invitrogen, 65601; 20 μl of slurry and sample). After incubation, the beads were isolated with a magnetic stand and washed twice with 1 ml of IP buffer, followed by once with 1 ml of HPLC-grade water. The eluted RNA was then digested by nucleoside digestion mix (NEB, M0649S). The digested nucleosides were subjected to LC–MS analysis.

The experiment with the 60-nt probe was performed as described above. After the time points, the liquid was subjected to a Monarch Spin RNA cleanup kit (10 μg) (NEB, T2030L), eluted with 17 μl of water and digested overnight as described above.

The nucleosides were analyzed by LC–MS-based multiple reaction monitoring (MRM) (Agilent Technologies 6460 or 6470 triple-quadrupole instrument). MS analysis was performed using positive-mode ESI with the following parameters: drying gas temperature, 350 °C; drying gas flow, 9 l min^−1^; nebulizer pressure, 45 psi; sheath gas temperature, 375 °C; sheath gas flow, 10 l min^−1^; fragmentor voltage, 135 V; capillary voltage, 4.5 kV. The separation of the analyte was achieved using a Gemini C18 column (50 mm × 4.6 mm, 5 μm; Phenomenex) coupled to a guard column (Gemini C18, 4 × 3 mm). The LC solvents were as follows: solvent A, 5 mM ammonium acetate; solvent B, acetonitrile. The LC gradient following injection increased from 0% to 5% B at 0.5 ml min^−1^ over 5 min, then increased to 90% B at 0.5 ml min^−1^ over 10 min and finally decreased to 0% B at 0.5 ml min^−1^ over 5 min. m^6^A (282.1 *m/*z→150.1 *m/*z), adenosine (268.1 *m/*z→136.0 *m/*z). Adenosine and m^6^A concentrations were calculated from external calibration curves.

### In vitro MT assay (MTase-Glo)

The rRNA probe (12 nt; Fig. 5b) was synthesized by IDT and the assay was performed according to the MTase-Glo MT assay manual.

Purified 20 μΜ WT-TRMT112:His–METTL5 or TRMT112-C100A:His–METTL5 complexes were incubated with 40 μΜ FWG-33B in reaction buffer (total volume: 20 μl; 50 mM Tris-HCl pH 7.5, 200 mM NaCl, 1 mM DTT and 5% DMSO) for 30 min at room temperature. The reaction mixtures were diluted tenfold with 180 μl of reaction buffer. Then, 2 μl of the diluted reaction mixtures (10×, final concentration: 0.2 μM) were added to 18 μl of the substrate assay (final concentrations: 20 mM Tris-HCl pH 7.5, 50 mM NaCl, 1 mM DTT and 0.5% DMSO) containing varying amounts of SAM or rRNA in a 96-well PCR plate (BrandTech, 781368). For the saturating SAM condition, we used 40 μΜ SAM. For the saturating rRNA condition, we used 50 μΜ 12-nt probe. Each enzymatic assay was performed in duplicate or triplicate from a single treatment (referred to as technical replicates). After incubating the substrate assay for 20 min on a rocker (50 rpm) at room temperature, 5 μl of 0.5% trifluoroacetic acid (TFA; final concentration: 0.1%) was added. Then, 10 μl of the quenched reaction mixtures or SAH standard (prepared in water) was transferred to a white 384-well microplate (Greiner, 784075). Next, 2 μl of prepared 6× MTase-Glo reagent was added to each well, followed by mixing and incubation at room temperature for 30 min. Then, 12 μl of MTase-Glo detection solution was added to each well, followed by mixing and incubation at room temperature for 30 min. Luminescence was measured on a Clariostar plate reader. SAH formation was quantified using a linear SAH calibration curve. The data were fitted using Michaelis–Menten fitting across all replicates (modified to symmetrical confidence intervals (CIs) to compute *k*_cat_/*K*_m_) in GraphPad Prism (version 10.4.1).

### Detection of SAM from denatured TRMT112:METTL5 by LC–MS

WT-TRMT112:His–METTL5 was precipitated by the addition of 1 ml of cold methanol to the purified complex (19 μl at 147 μM) on ice. After 1 h, the mixture was centrifuged at 21,000*g* for 10 min. The supernatant was transferred to a new tube and dried under a flow of nitrogen gas. HPLC-grade water (52 μl) was added and subjected to LC–MS analysis.

The sample was analyzed by LC–MS-based MRM (Agilent Technologies 6470 triple-quadrupole instrument). MS analysis was performed using positive-mode ESI with the following parameters: drying gas temperature, 350 °C; drying gas flow, 9 l min^−1^; nebulizer pressure, 45 psi; sheath gas temperature, 375 °C; sheath gas flow, 10 l min^−1^; fragmentor voltage, 135 V; capillary voltage, 5.3 kV. The separation of the analyte was achieved using a Gemini C18 column (50 mm × 4.6 mm, 5 μm; Phenomenex) coupled to a guard column (Gemini C18, 4 × 3 mm). The LC solvents were as follows: solvent A, 5 mM ammonium acetate; solvent B, acetonitrile. The LC gradient following injection increased from 0% to 10% B at 0.6 ml min^−1^ over 1 min, then increased to 90% B at 0.6 ml min^−1^ over 3 min, then increased to 100% B at 0.6 ml min^−1^ over 2 min and finally decreased to 0% B at 0.6 ml min^−1^ over 1 min. SAM (398.99 *m/*z→136.1 *m/*z). The chromatogram and spectrum were compared with SAM standard.

### Statistics and reproducibility

Statistical analyses and data visualization in this paper were performed using GraphPad Prism (version 10.4.1). To compare group means, we performed multiple two-sided, unpaired *t*-tests between group pairs, using the Holm–Šídák method to correct for multiple comparisons; reported *P* values are adjusted *P* values. For experiments comparing multiple treatments to a single treatment group, we used a one-way analysis of variance (ANOVA) followed by Dunnett’s post hoc test.

### Reporting summary

Further information on research design is available in the Nature Portfolio Reporting Summary linked to this article.

## Online content

Any methods, additional references, Nature Portfolio reporting summaries, source data, extended data, supplementary information, acknowledgements, peer review information; details of author contributions and competing interests; and statements of data and code availability are available at 10.1038/s41589-025-02099-5.

## Supplementary information

Supplementary Information Supplementary Tables 1–9, Figs. 1–3 and synthetic chemistry methods.

Reporting Summary

Supplementary Data 1 Unprocessed proteomic data related to Table 1, Figs. 1c, 2f and 3g,h, and Extended Data Figs. 4b, 5e,g, 6g and 7g.

## Source data

Source Data Fig. 1 Statistical source data.

Source Data Fig. 1 Unprocessed western blots and/or gels.

Source Data Fig. 2 Statistical source data.

Source Data Fig. 2 Unprocessed western blots and/or gels.

Source Data Fig. 3 Statistical source data.

Source Data Fig. 3 Unprocessed western blots and/or gels.

Source Data Fig. 4 Unprocessed western blots and/or gels.

Source Data Fig. 5 Statistical source data.

Source Data Extended Data Fig. 2 Statistical source data.

Source Data Extended Data Fig. 4 Statistical source data.

Source Data Extended Data Fig. 4 Unprocessed western blots and/or gels.

Source Data Extended Data Fig. 5 Statistical source data.

Source Data Extended Data Fig. 5 Unprocessed western blots and/or gels.

Source Data Extended Data Fig. 6 Statistical source data.

Source Data Extended Data Fig. 6 Unprocessed western blots and/or gels.

Source Data Extended Data Fig. 7 Statistical source data.

Source Data Extended Data Fig. 7 Unprocessed western blots and/or gels.

Source Data Extended Data Fig. 8 Statistical source data.

Source Data Extended Data Fig. 8 Unprocessed western blots and/or gels.

Source Data Extended Data Fig. 10 Statistical source data.

## Data Availability

Proteomic data are available through ProteomeXchange^76^ with identifier PXD063358. The atomic coordinates and structure factors were deposited to the Protein Data Bank under accession code 9OHL. Source data are provided with this paper.
