# Supplementary material for: Complexoform-restricted covalent TRMT112 ligands that allosterically agonize METTL5
Source: Nat Chem Biol. 2026 Jan 8;22(5):770–82. doi: 10.1038/s41589-025-02099-5 (PMC13128453; doi:10.1038/s41589-025-02099-5)
Supplement: Supplementary file 2 — Reporting Summary [file 41589_2025_2099_MOESM2_ESM.pdf]

nature portfolio

Corresponding author(s): Benjamin F. Cravatt

Last updated by author(s): Oct 30, 2025

Reporting Summary

Nature Portfolio wishes to improve the reproducibility of the work that we publish. This form provides structure for consistency and transparency in reporting. For further information on Nature Portfolio policies, see our Editorial Policies and the Editorial Policy Checklist.

Statistics

For all statistical analyses, confirm that the following items are present in the figure legend, table legend, main text, or Methods section.

n/a	Confirmed	
☐	☒	The exact sample size (*n*) for each experimental group/condition, given as a discrete number and unit of measurement
☐	☒	A statement on whether measurements were taken from distinct samples or whether the same sample was measured repeatedly
☐	☒	The statistical test(s) used AND whether they are one- or two-sided<br>*Only common tests should be described solely by name; describe more complex techniques in the Methods section.*
☒	☐	A description of all covariates tested
☐	☒	A description of any assumptions or corrections, such as tests of normality and adjustment for multiple comparisons
☐	☒	A full description of the statistical parameters including central tendency (e.g. means) or other basic estimates (e.g. regression coefficient) AND variation (e.g. standard deviation) or associated estimates of uncertainty (e.g. confidence intervals)
☐	☒	For null hypothesis testing, the test statistic (e.g. *F*, *t*, *r*) with confidence intervals, effect sizes, degrees of freedom and *P* value noted<br>*Give P values as exact values whenever suitable.*
☒	☐	For Bayesian analysis, information on the choice of priors and Markov chain Monte Carlo settings
☒	☐	For hierarchical and complex designs, identification of the appropriate level for tests and full reporting of outcomes
☐	☒	Estimates of effect sizes (e.g. Cohen's *d*, Pearson's *r*), indicating how they were calculated

Our web collection on statistics for biologists contains articles on many of the points above.

Software and code

Policy information about availability of computer code

Data collection	Thermo Scientific Xcalibur software version 2.2 software was used to collect raw proteomics data. BioRad Image Lab software version 6.1 was used for immunoblot and fluorescent gel imaging. MRM data for METTL5 methyltransferase assay was acquired on Agilent Technologies 6460 or 6470 Triple Quad instrument with MassHunter Workstation LC/MS Acquisition Console version 10.0. NMR spectra were recorded on Bruker AVIII HD 600 MHz NMR (equipped with 5 mm CPQCI and 1.7 mm CPTCI CryoProbes), Bruker AVIII HD 600 MHz NMR (equipped with 5 mm CPDCH CryoProbe), Bruker AV NEO 500 MHz NMR (equipped with 5 mm BBFO Probe) or Bruker AV NEO 399 MHz NMR instruments. Mass measurements using high-resolution mass spectrometry (HRMS) were performed on a Waters Xevo G2-XS TOF instrument. Chiral SFC (supercritical fluid chromatography) separations were conducted on a Waters Acquity UPC2 system using Daicel columns as stationary phase and Waters Empower software. Diffraction data were collected on Advanced Light Source Beamline 5.0.2 and processed with XDS.
Data analysis	The Integrated Proteomics Pipeline (IP2, version 6.7.1) was used to process quantitative proteomics data using the ProLuCID program. BioRad Image Lab software version 6.1 was used for analysis of immunoblot and fluorescent gel imaging. NMR spectra were processed with MestReNova v15.1.0. The crystal structure was determined by molecular replacement in Phaser and was refined using iterative rounds of refinement in REFMAC5 with manual inspection and model building in COOT. Schrödinger Maestro (version 13.2.128, MMshare Version 5.8.128, Release 2022-2, Platform Darwin-x86_64) and Chimera-X v.1.9 were used for the analysis of crystal structures. Statistical analysis was performed using GraphPad Prism v.10 (v.10.4.1).

For manuscripts utilizing custom algorithms or software that are central to the research but not yet described in published literature, software must be made available to editors and reviewers. We strongly encourage code deposition in a community repository (e.g. GitHub). See the Nature Portfolio guidelines for submitting code & software for further information.

Data

Policy information about availability of data

All manuscripts must include a data availability statement. This statement should provide the following information, where applicable:

- Accession codes, unique identifiers, or web links for publicly available datasets
- A description of any restrictions on data availability
- For clinical datasets or third party data, please ensure that the statement adheres to our policy

Proteomic data are available via ProteomeXchange with identifier PXD063358. The atomic coordinates and structure factors have been deposited in the Protein Data Bank, www.pdb.org (PDB ID code 9OHL).

Research involving human participants, their data, or biological material

Policy information about studies with human participants or human data. See also policy information about sex, gender (identity/presentation), and sexual orientation and race, ethnicity and racism.

Reporting on sex and gender	N/A
Reporting on race, ethnicity, or other socially relevant groupings	N/A
Population characteristics	N/A
Recruitment	N/A
Ethics oversight	N/A

Note that full information on the approval of the study protocol must also be provided in the manuscript.

Field-specific reporting

Please select the one below that is the best fit for your research. If you are not sure, read the appropriate sections before making your selection.

☒ Life sciences ☐ Behavioural & social sciences ☐ Ecological, evolutionary & environmental sciences

For a reference copy of the document with all sections, see nature.com/documents/nr-reporting-summary-flat.pdf

Life sciences study design

All studies must disclose on these points even when the disclosure is negative.

Sample size	The sample sizes used in different experiments were empirically determined. At least three biological replicates is required when testing statistical significance.
Data exclusions	As described in the Biology Methods, we excluded proteomic data that failed to pass thresholds for signal intensity, signal to noise, isolation purity, and coefficients of variability. No other data were excluded.
Replication	The independent biological replicates were labeled in the figure legends.
Randomization	Human cancer cell lines were cultured under identical conditions and the experiment conditions were randomly assigned.
Blinding	Human cancer cell lines were cultured under identical conditions and blinding was not used.

Reporting for specific materials, systems and methods

We require information from authors about some types of materials, experimental systems and methods used in many studies. Here, indicate whether each material, system or method listed is relevant to your study. If you are not sure if a list item applies to your research, read the appropriate section before selecting a response.

Materials & experimental systems

n/a	Involved in the study	
☐	☒	Antibodies
☐	☒	Eukaryotic cell lines
☒	☐	Palaeontology and archaeology
☒	☐	Animals and other organisms
☒	☐	Clinical data
☒	☐	Dual use research of concern
☒	☐	Plants

Methods

n/a	Involved in the study	
☒	☐	ChIP-seq
☒	☐	Flow cytometry
☒	☐	MRI-based neuroimaging

Antibodies

Antibodies used

1) FLAG® (HRP) (M2) (Sigma-Aldrich: A8592; IB 1:5,000 dilution)
2) β-Actin Antibody (C4) (Santa Cruz: sc-47778; IB 1:5,000 dilution)
3) GAPDH (HRP) (D16H11) (Cell signaling: 8884; IB 1:2,000 dilution)
4) HA (HRP) (6E2) (Cell signaling: 2999; IB 1:2,000 dilution)
5) Vinculin (E1E9V) (HRP) (18799; IB 1:2,000 dilution)
6) TRMT112 (F-7) (Santa Cruz: sc-398481; IB 1:1,000 dilution)
7) METTL5 (Proteintech: 16791-1-AP; IB 1:2,000 dilution)
8) BUD23 (Proteintech: 28192-1-AP; IB 1:3,000 dilution)
9) THUMPD3 (Proteintech: 19807-1-AP; IB 1:2,000 dilution)
10) N6AMT1 (Proteintech: 16211-1-AP; IB 1:1,000 dilution)
11) TRMT11 (Proteintech: 17555-1-AP; IB 1:1,000 dilution)
12) THUMPD2 (D1) (Santa Cruz: sc-393018; IB 1:500 dilution)
13) ALKBH8 (Sigma-Aldrich: HPA061514; IB 1:500 dilution)
14) anti-mouse IgG (HRP) (Cell signaling: 7076; IB 1:2,000 dilution)
15) anti-rabbit IgG (HRP) (Santa Cruz: sc-2357; IB 1:5,000 dilution)

Validation

All antibodies are commercially available and validated by manufacturers.

Eukaryotic cell lines

Policy information about cell lines and Sex and Gender in Research

Cell line source(s)

Ramos (ATCC, CRL-1596), HEK293T (ATCC, CRL-3216), Lenti-X (Takara, #632180), HCT116 (ATCC, CCL-247) and 22Rv1 (ATCC, CRL-2505) were used in this study.

Authentication

All cell lines were authenticated by short tandem repeat loci (STRs) profiling by vendors.

Mycoplasma contamination

All cell lines were routinely inspected for mycoplasma contamination.

Commonly misidentified lines
(See ICLAC register)

No commonly misidentified cell lines were used in this study.

Plants

Seed stocks

Report on the source of all seed stocks or other plant material used. If applicable, state the seed stock centre and catalogue number. If plant specimens were collected from the field, describe the collection location, date and sampling procedures.

Novel plant genotypes

Describe the methods by which all novel plant genotypes were produced. This includes those generated by transgenic approaches, gene editing, chemical/radiation-based mutagenesis and hybridization. For transgenic lines, describe the transformation method, the number of independent lines analyzed and the generation upon which experiments were performed. For gene-edited lines, describe the editor used, the endogenous sequence targeted for editing, the targeting guide RNA sequence (if applicable) and how the editor was applied.

Authentication

Describe any authentication procedures for each seed stock used or novel genotype generated. Describe any experiments used to assess the effect of a mutation and, where applicable, how potential secondary effects (e.g. second site T-DNA insertions, mosiacism, off-target gene editing) were examined.
