# Supplementary material for: Complexoform-restricted covalent TRMT112 ligands that allosterically agonize METTL5
Source: Nat Chem Biol. 2026 Jan 8;22(5):770–82. doi: 10.1038/s41589-025-02099-5 (PMC13128453; doi:10.1038/s41589-025-02099-5)
Supplement: Supplementary file 16 — Unprocessed western blots and/or gels. [file 41589_2025_2099_MOESM16_ESM.pdf]

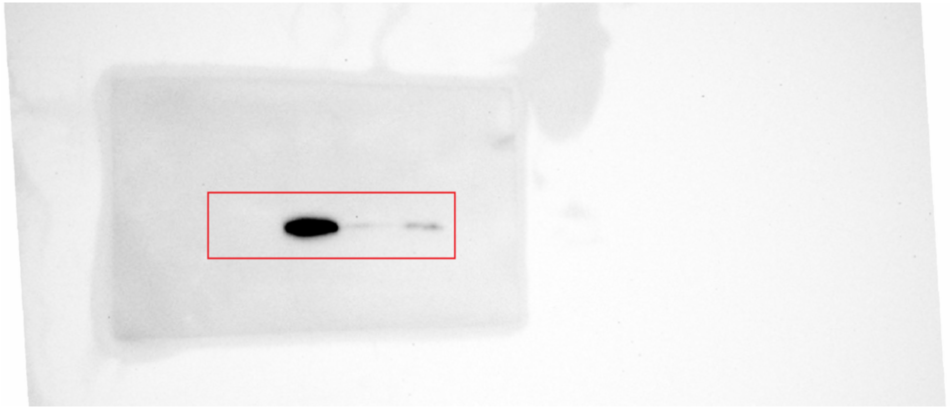

Uncropped image of Chemiluminescence scan in reference to Extended Data Fig. 5f (IB: FLAG).

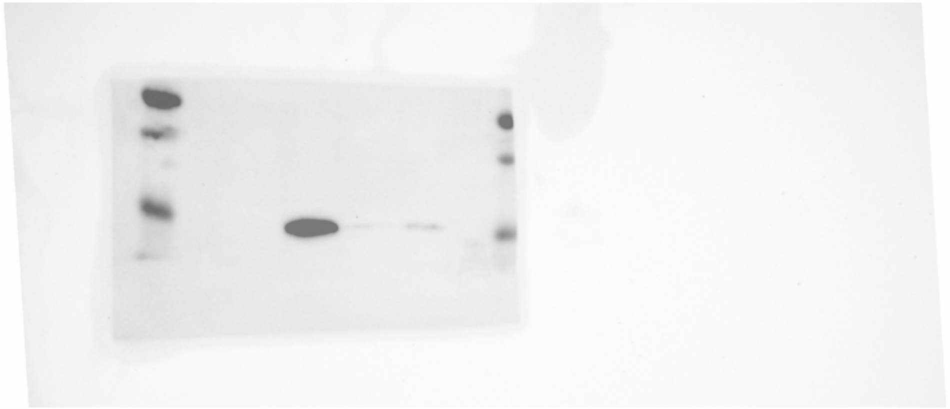

Uncropped composite image of Cy5 and Chemiluminescence scan in reference to Extended Data Fig. 5f (IB: FLAG).

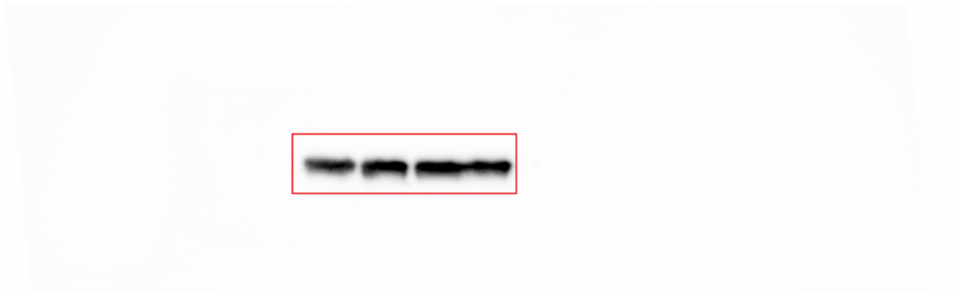

Uncropped image of Chemiluminescence scan in reference to Extended Data Fig. 5f (IB: β-actin).

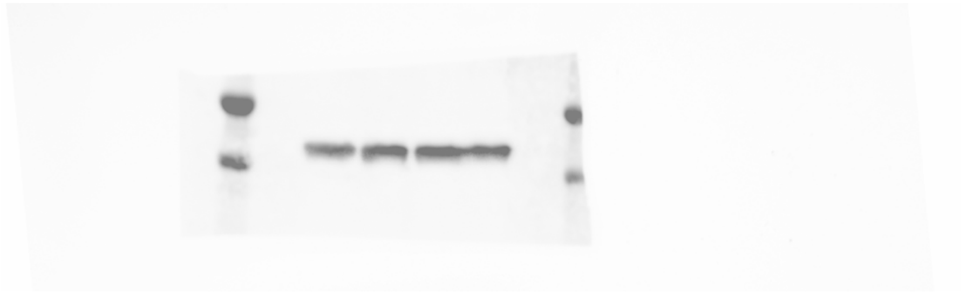

Uncropped composite image of Cy5 and Chemiluminescence scan in reference to Extended Data Fig. 5f (IB: β-actin).
