# Supplementary material for: Complexoform-restricted covalent TRMT112 ligands that allosterically agonize METTL5
Source: Nat Chem Biol. 2026 Jan 8;22(5):770–82. doi: 10.1038/s41589-025-02099-5 (PMC13128453; doi:10.1038/s41589-025-02099-5)
Supplement: Supplementary file 22 — Unprocessed western blots and/or gels. [file 41589_2025_2099_MOESM22_ESM.pdf]

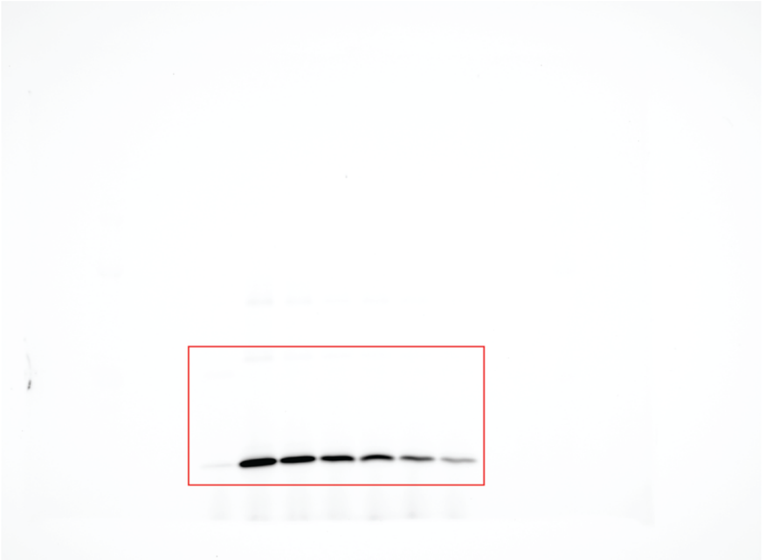

Uncropped image of Rhodamine scan in reference to Extended Data Fig. 8b (ABPP).

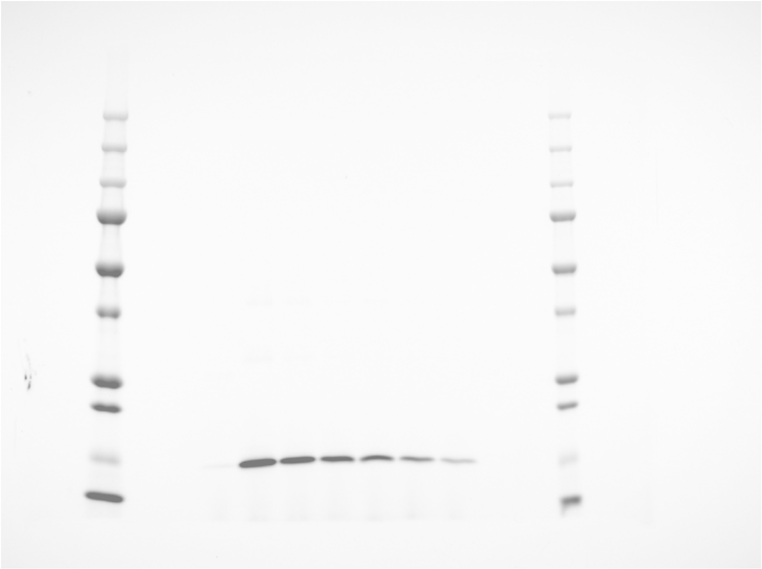

Uncropped composite image of Cy5 and Rhodamine scan in reference to Extended Data Fig. 8b (ABPP).

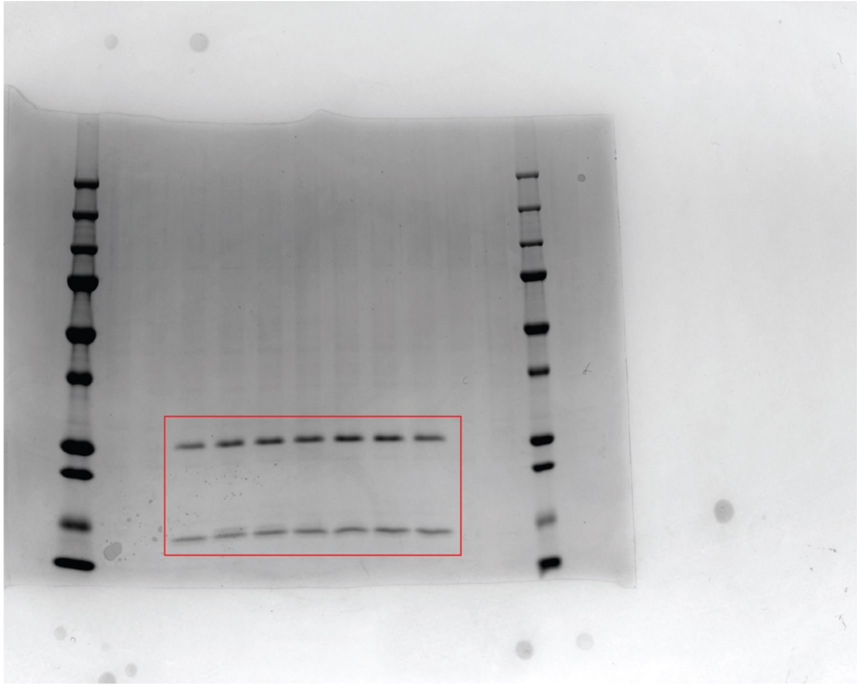

Uncropped composite Coomassie scan in reference to Extended Data Fig. 8b

(Coomassie).

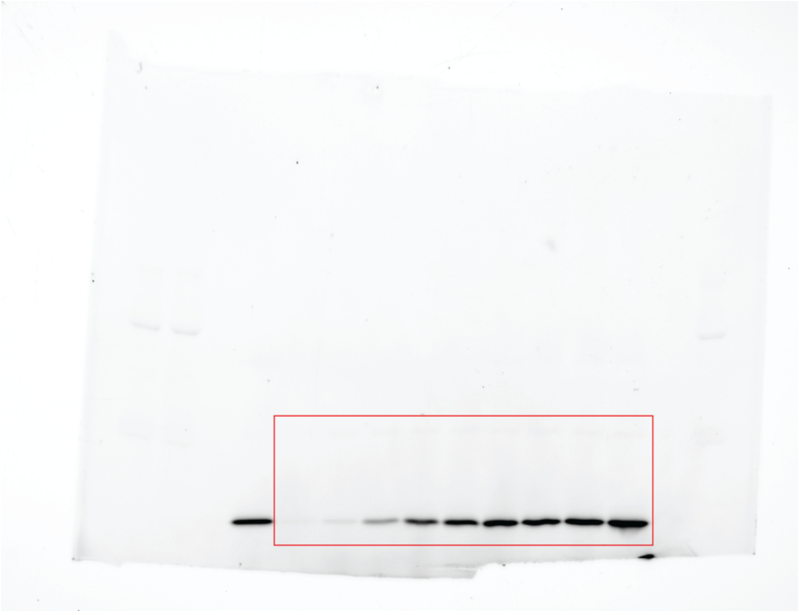

Uncropped image of Rhodamine scan in reference to Extended Data Fig. 8d (ABPP).

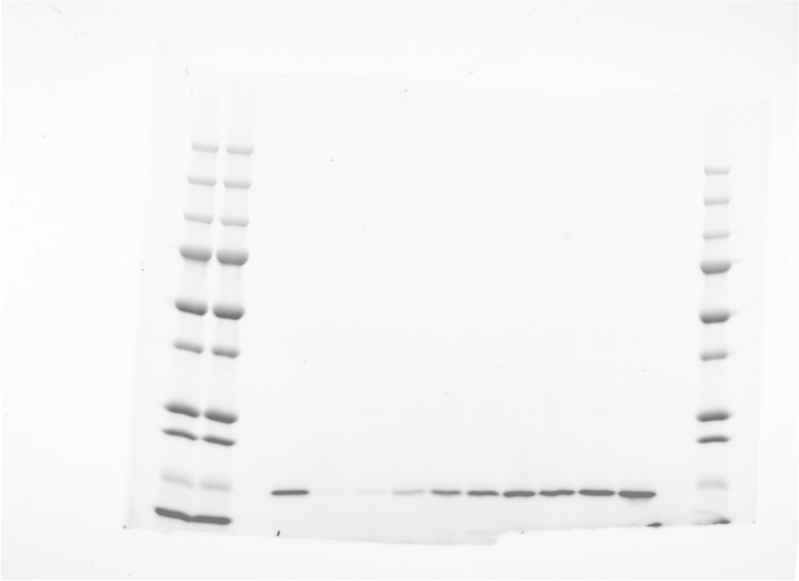

Uncropped composite image of Cy5 and Rhodamine scan in reference to Extended Data Fig. 8d (ABPP).

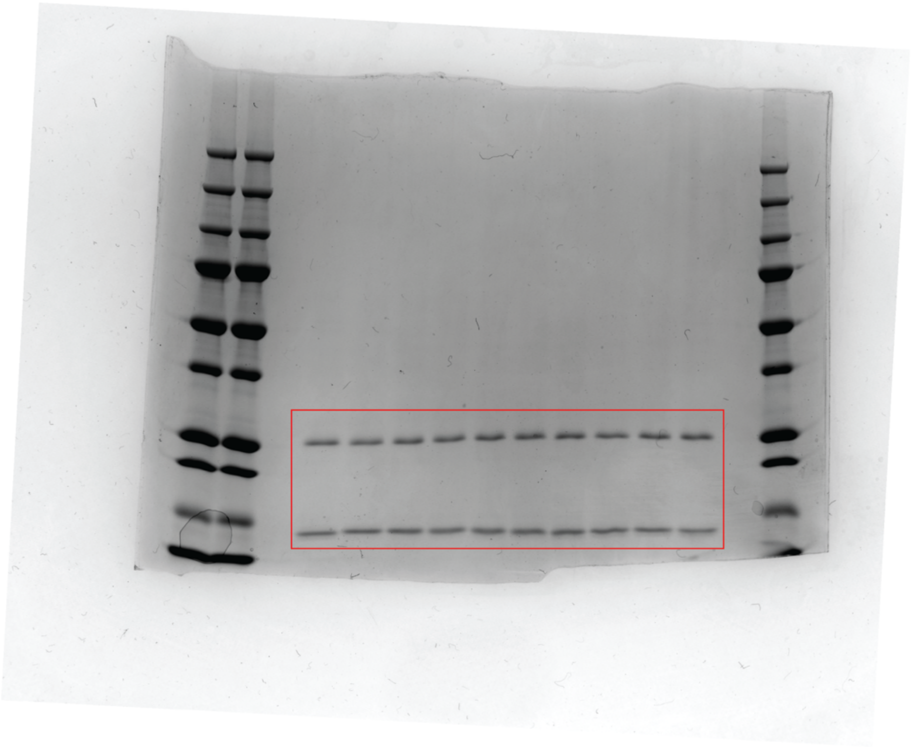

Uncropped composite Coomassie scan in reference to Extended Data Fig. 8d

(Coomassie).

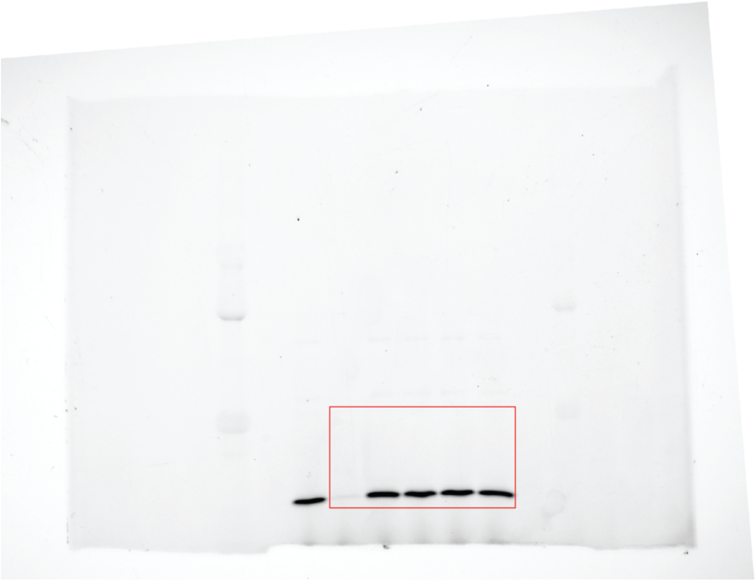

Uncropped image of Rhodamine scan in reference to Extended Data Fig. 8f (ABPP).

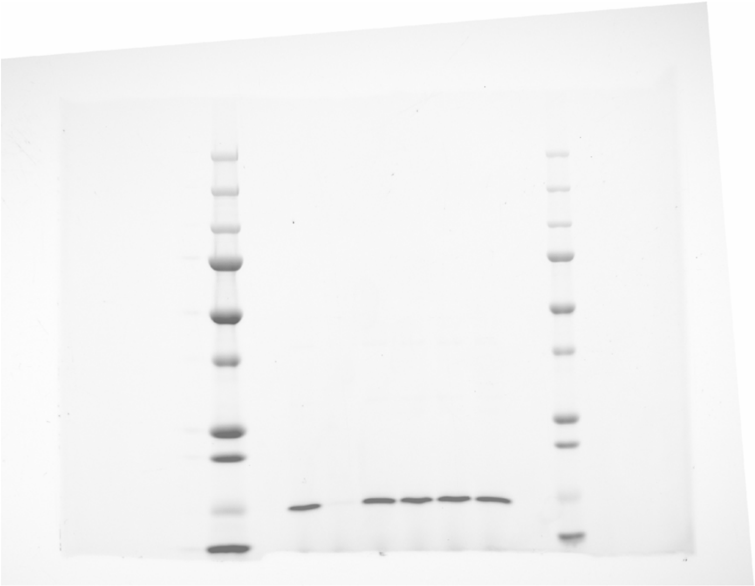

Uncropped composite image of Cy5 and Rhodamine scan in reference to Extended Data Fig. 8f (ABPP).

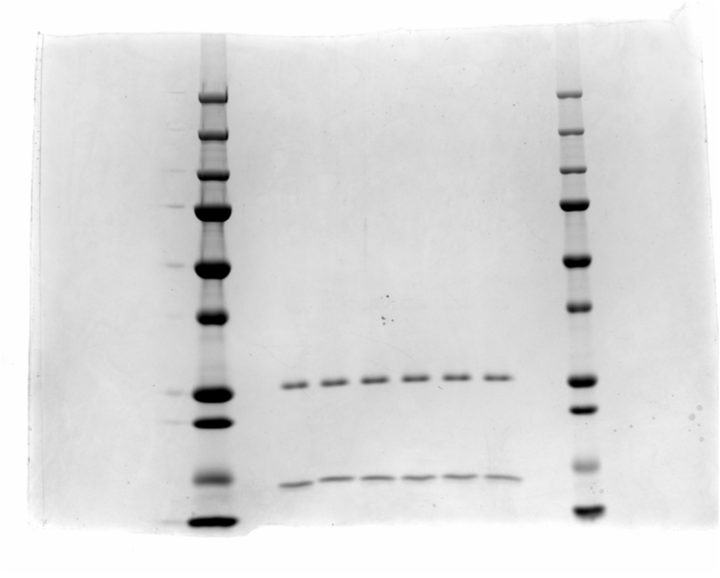

Uncropped composite Coomassie scan in reference to Extended Data Fig. 8f (Coomassie).
